# Mild kidney dysfunction affects the predictive accuracy of blood‐based biomarkers for neuropsychological and neuroimaging outcomes over a 9 year follow‐up period

**DOI:** 10.1002/alz.70651

**Published:** 2025-09-19

**Authors:** Corey J. Bolton, Panpan Zhang, Devika Nair, Dandan Liu, L. Taylor Davis, Kimberly R. Pechman, Niranjana Shashikumar, Sydney Wilhoite, Dominic Roby, Carlie Beeson, Haley Komorowski, Katherine A. Gifford, Timothy J. Hohman, Kaj Blennow, Henrik Zetterberg, Angela L. Jefferson

**Affiliations:** ^1^ Vanderbilt Memory and Alzheimer's Center Vanderbilt University School of Medicine Nashville Tennessee USA; ^2^ Department of Medicine Vanderbilt University Medical Center Nashville Tennessee USA; ^3^ Department of Biostatistics Vanderbilt University Medical Center Nashville Tennessee USA; ^4^ Tennessee Valley Veterans Affairs Health System Nashville Tennessee USA; ^5^ Veteran Wellbeing through Innovation Systems Science and Experience in Learning Health Systems (VETWISE‐LHS) Center of Innovation Nashville Tennessee USA; ^6^ Department of Neurology Vanderbilt University Medical Center Nashville Tennessee USA; ^7^ Department of Radiology and Radiological Sciences Vanderbilt University Medical Center Nashville Tennessee USA; ^8^ Department of Anatomy and Neurobiology Boston University Chobanian & Avedisian School of Medicine Boston Massachusetts USA; ^9^ Department of Psychiatry and Neurochemistry Institute of Neuroscience and Physiology The Sahlgrenska Academy at University of Gothenburg Gothenburg Sweden; ^10^ Clinical Neurochemistry Lab Sahlgrenska University Hospital Gothenburg Sweden; ^11^ Department of Molecular Neuroscience UCL Institute of Neurology Queen Square London UK; ^12^ UK Dementia Research Institute at UCL London UK; ^13^ Hong Kong Center for Neurodegenerative Diseases Hong Kong Science Park Hong Kong China; ^14^ Wisconsin Alzheimer's Disease Research Center University of Wisconsin School of Medicine and Public Health University of Wisconsin–Madison Madison Wisconsin USA

**Keywords:** Alzheimer's disease, biomarkers, blood‐based biomarkers, kidney disease, kidney function

## Abstract

**INTRODUCTION:**

The impact of chronic kidney disease (CKD) on Alzheimer's disease (AD) plasma biomarkers is poorly understood. We tested whether kidney function decline affects the predictive accuracy of plasma biomarkers on neuropsychological or neuroimaging outcomes.

**METHODS:**

Three hundred thirty‐three non‐demented older adults were included. Linear regressions related plasma glial fibrillary acidic protein, neurofilament light chain (NfL), amyloid beta 42, and phosphorylated tau_231_, with a blood‐based biomarker x estimated glomerular filtration rate (eGFR) interaction term, to cross‐sectional and longitudinal (mean follow‐up time = 6.4 ± 2.8 years) neuropsychological and neuroimaging outcomes.

**RESULTS:**

Plasma NfL interacted with eGFR on the longitudinal trajectory of nearly all neuropsychological outcomes (*p* values < 0.02) and several neuroimaging outcomes (*p* values < 0.02). Associations were stronger in individuals with no CKD/stage 1 and stage 2 CKD, while associations were weaker or not significant in individuals with stage 3 CKD.

**DISCUSSION:**

Among older adults free of severe CKD, the ability of plasma NfL to predict key AD‐related biomarker outcomes was moderated by renal function.

**Highlights:**

- Reduced kidney function was associated with increased blood biomarker levels.
- The predictive accuracy of neurofilament light chain was reduced in participants with kidney dysfunction.
- Even mild kidney dysfunction can affect blood biomarkers of Alzheimer's disease.

## 1 INTRODUCTION

Cerebrospinal fluid (CSF) and positron emission tomography measurements of amyloid beta (Aβ) and tau can detect Alzheimer's disease (AD) pathology in vivo; however, these methods are not widely accessible. Novel blood‐based biomarkers of AD pathology and concomitant neuropathological pathways are increasingly accessible and have the potential to improve the feasibility and scalability of AD diagnosis. Plasma‐based measures of Aβ,^1^ phosphorylated tau (p‐tau),^2^ glial fibrillary acidic protein (GFAP),^3^ and neurofilament light chain (NfL)^4^ predict key AD‐related clinical outcomes, However, there are challenges to interpreting biomarker levels in the blood, as some chronic medical conditions can affect the clearance of circulating proteins, and most studies thus far have been in relatively healthy cohorts and have not considered medical comorbidities that are common in older adults that may affect blood protein levels.^5^ Reduced kidney function is one common medical comorbidity in aging that affects the clearance of various blood‐based biomarkers,^6^, ^7^ and understanding the impact of kidney function on blood‐based biomarkers has been highlighted as a key research priority in recent appropriate use guidelines for blood biomarkers in AD.^5^ This issue represents a critical knowledge gap that requires attention prior to the widespread implementation of blood biomarkers for diagnosing AD in the clinical setting.

Chronic kidney disease (CKD) is prevalent in 38% of adults aged ≥ 65.^8^ CKD and estimated glomerular filtration rate (eGFR), a marker of kidney function, are associated with elevated levels of many plasma biomarkers, including NfL, p‐tau, total tau, Aβ_40_, and Aβ42, likely reflecting impaired clearance of these proteins from the blood.^6^, ^7^, ^9^ However, it is not known whether these changes in biomarker levels impact the diagnostic and prognostic utility of blood biomarkers in predicting relevant clinical changes.

To address this knowledge gap, this study aims to first replicate findings in the field demonstrating the ability of blood‐based biomarkers relevant to AD (GFAP, NfL, Aβ42, and p‐tau_231_) to predict cross‐sectional and longitudinal neuropsychological and neuroimaging outcomes reflective of cognitive decline and neurodegeneration. We then examine interactions between these blood‐based biomarkers and eGFR levels on each outcome to determine whether the predictive accuracy of blood‐based biomarkers varies across different levels of renal functioning (no CKD/stage 1 CKD, stage 2 CKD, stage 3 CKD). Based on past work showing kidney function–related changes in biomarker levels,^6^, ^7^, ^9^ we hypothesized that associations between blood‐based biomarkers and clinical outcomes would be modified by eGFR. This study will provide critical information regarding the performance of blood‐based biomarkers in individuals with a prevalent medical condition common in aging, thereby aiding in the clinical interpretation of biomarker results for the AD field.

## 2 METHODS

### 2.1 Study cohort

Participants were drawn from the Vanderbilt Memory and Aging project,^10^ a longitudinal, observational study investigating vascular and brain health among aging adults free of dementia at study entry. Participants underwent a medical history review, clinical interview, and neuropsychological assessment to determine study eligibility, and their cognitive status was determined by a consensus panel. Cognitive status was defined as cognitively unimpaired (CU), early mild cognitive impairment (MCI),^11^ or MCI.^12^ Participants were recruited from the community and met the following inclusion criteria: English fluency, ≥ 60 years old, availability of a reliable study partner, and adequate auditory and visual skills to complete assessments. Participants were excluded for major psychiatric illness, metal screening contraindications for magnetic resonance imaging (MRI), a history of other neurological illness, heart failure, terminal illness, or significant head injury with a loss of consciousness for > 5 minutes. Participants completed a comprehensive evaluation upon study enrollment (2012–2014), including (but not limited to) fasting blood draw; multimodal brain MRI; neuropsychological assessment; clinical interview, including Clinical Dementia Rating scale;^13^ study partner reported and self‐reported functional abilities questionnaires; and optional lumbar puncture. All procedures were repeated at 18 month (2014–2016), 3 year (2015–2018), 5 year (2017–2020), 7 year (2019–2022), 9 year (2022–2024), and 11 year (2024‐ongoing) follow‐up visits. Participants were excluded from analyses for missing plasma biomarker data, covariate data, or for not having at least one outcome of interest.

The institutional review board of Vanderbilt University Medical Center approved the protocol. Before data collection, written informed consent was obtained from each participant. Data and protocols are available for sharing and may be obtained by contacting the corresponding author.

**RESEARCH IN CONTEXT**

1. **Systematic review**: The authors reviewed the literature using traditional (e.g., PubMed) sources and meeting abstracts and presentations. There have been several recent publications examining the effect of medical conditions on plasma biomarkers. These relevant publications are appropriately cited.
2. **Interpretation**: Our findings demonstrated that the predictive accuracy of some plasma biomarkers of Alzheimer's disease and concomitant pathologies for longitudinal changes in neuropsychological and neuroimaging outcomes was affected by mild kidney dysfunction. These findings demonstrate the importance of considering an individual's kidney function when interpreting blood biomarker results.
3. **Future directions**: This article provides key insights into the effect of kidney function on plasma biomarker performance in individuals without severe kidney dysfunction. Future studies should investigate the effects of kidney function on plasma biomarker performance in individuals with more significant kidney disease.

### 2.2 Fluid collection and biochemical analyses

Participants underwent a fasting venous blood draw at study entry. Clinical lab work, including quantification of creatinine, was completed on site immediately after blood draw. eGFR was quantified using the CKD Epidemiology Collaboration (CKD‐EPI) formula based on age, sex, and serum creatinine level.^14^ From the same blood draw, plasma was isolated from ethylenediaminetetraacetic acid whole blood via centrifugation (2000 × g at 4°C for 15 minutes) and within 3 hours (per Standardization of Alzheimer's Blood Biomarkers working group recommendations)^15^ was aliquoted into 0.5 mL polypropylene tubes and stored at –80°C until further analysis. All plasma biomarkers were batch analyzed by certified laboratory technicians at the Clinical Neurochemistry Laboratory at Sahlgrenska University Hospital, Mölndal, Sweden in 2022 (≈ 8–10 years after baseline blood collection). All laboratory technicians were blinded to clinical information. Plasma GFAP, NfL, Aβ42, and Aβ40 concentrations were measured using commercially available assays (Quanterix Neurology 4‐plex E) on the Simoa HD‐X (Quanterix) analyzer. Plasma p‐tau231 concentration was measured using an in‐house Simoa assay as previously described.^16^ For each biomarker, intraassay coefficients of variation were < 10%.

### 2.3 Neuropsychological assessment

Participants completed a comprehensive neuropsychological protocol assessing language, information processing speed, visuospatial skills, executive function, and episodic memory at each time point (see Table 1 for individual measures). To reduce multiple comparisons, executive function and memory composite *z* scores were calculated from item‐level data using latent variable modeling as previously described.^17^

**TABLE 1:** Participant characteristics at study entry stratified by CKD stage.

	Combined (*n* = 333)	No CKD/ Stage 1 (*n* = 57)	Stage 2 (*n* = 217)	Stage 3 (*n* = 59)	*p* value^b^
Age, years	73 ± 7.3	70 ± 6.9	73 ± 6.8	77 ± 7.8	**<0.001**
Sex, % male	59	47	61	63	0.14
Education, years	16 ± 2.7	16 ± 2.5	16 ± 2.6	16 ± 2.8	0.27
Race, % non‐Hispanic White	86	96	86	80	**0.03**
*APOE* ε4, % carrier	35	37	33	39	0.62
Cognitive status, % MCI	40	39	38	46	0.73
MoCA, total	25.3 ± 3	26.2 ± 3	25.4 ±3	24.2 ± 4	**0.01**
FSRP score^a^	13 ± 4.2	11 ± 4	12 ± 4.3	14 ± 3.6	**<0.001**
CSF amyloid status, % positive	46	42	44	65	0.20
eGFR, mL/minute/1.73m^2^	74.1 ± 16	98.6 ± 8.1	73.9 ± 8.2	51.2 ± 6.3	**<0.001**
Plasma GFAP, pg/mL	180 ± 99	170 ± 104	170 ± 89	230 ± 120	**0.002**
Plasma NfL, pg/mL	30 ± 21	25 ± 13	27 ± 14	46 ±36	**<0.001**
Plasma Aβ42, pg/mL	5.0 ± 2.4	4.5 ± 1.6	4.9 ± 2.5	5.8 ± 2.7	**0.001**
Plasma p‐tau_231_, pg/mL	6.8 ± 4.2	6.4 ± 3.8	6.4 ± 3.8	8.5 ± 5.5	**0.01**
**Neuropsychological outcomes**					
Boston Naming Test, total	26.8 ± 3	27.4 ± 3	27.0 ± 3	25.5 ± 4	**0.01**
Animal Fluency, total	19.0 ± 5	19.6 ± 5	19.0 ± 5	18.4 ± 6	0.37
Number Sequencing, seconds	43 ± 20	37 ± 13	43 ± 21	48 ± 21	**0.002**
Digit Symbol Coding, total	53 ± 13	56 ± 13	53 ± 12	48 ± 21	**<0.001**
HVOT, total	24.4 ± 3	24.7 ± 3	24.8 ± 3	22.9 ± 4	**<0.001**
Executive function composite, *z*	0.01 ± 0.9	0.25 ± 0.8	0.037 ± 0.9	−0.31 ± 1.1	**0.01**
Episodic memory composite, *z*	−0.002 ± 1.0	0.20 ± 1.0	0.02 ± 1.0	−0.30 ± 1.0	**0.02**
**Gray matter MRI outcomes**					
AD signature cortical thickness, mm^2^	2.4 ± 0.1	2.5 ± 0.1	2.4 ± 0.1	2.4 ± 0.1	**0.01**
Frontal lobe gray matter, cm^3^	217.0 ± 31	210.7 ± 26	217.2 ± 31	222.3 ± 34	0.25
Temporal lobe gray matter, cm^3^	130.8 ± 16	127.3 ± 15	131.4 ± 16	131.9 ± 16	0.11
Parietal lobe gray matter, cm^3^	125.2 ± 17	121.9 ± 14	125.3 ± 17	127.5 ± 18	0.29
Occipital lobe gray matter, cm^3^	89.7 ± 10	88.3 ± 10	89.7 ± 11	91.3 ± 11	0.34
Hippocampal volume, cm^3^	7.0 ± 0.9	7.0 ± 0.9	7.1 ± 0.8	6.8 ± 1.0	0.06
Inferior lateral ventricle volume, cm^3^	2.0 ± 1.2	1.8 ± 1.3	1.9 ± 1.2	2.4 ± 1.4	**0.01**
**White matter MRI outcomes**					
Frontal WMH volume, cm^3^	7.0 ± 11	7.1 ± 15	6.7 ± 10	8.0 ± 10	0.11
Temporal WMH volume, cm^3^	0.6 ± 1.6	0.8 ± 2.8	0.6 ± 1.4	0.6 ± 0.8	0.10
Parietal WMH volume, cm^3^	3.3 ± 6.4	3.7 ± 8.9	3.3 ± 6.1	3.3 ± 4.7	0.22
Occipital WMH volume, cm^3^	2.6 ± 2.8	3.0 ± 3.8	2.5 ± 2.5	2.8 ± 2.5	0.77

*Note*: Values denoted as mean ± standard deviation or frequency. Bold font indicates *p* < 0.05.

Abbreviations: Aβ, amyloid beta; AD, Alzheimer's disease; *APOE*, apolipoprotein E; CKD, chronic kidney disease; CSF, cerebrospinal fluid; eGFR, estimated glomerular filtration rate; FSRP, Framingham Stroke Risk Profile; GFAP, glial fibrillary acidic protein; HVOT, Hooper Visual Organization Test; MCI, mild cognitive impairment; MoCA, Montreal Cognitive Assessment; MRI, magnetic resonance imaging; NfL, neurofilament light; p‐tau, phosphorylated tau; WMH, white matter hyperintensity.

^a^ A modified FSRP score excluded points assigned to age.

^b^ Kruskal–Wallis test was used for continuous variables, and Pearson chi‐squared test was used for categorical variables.

### 2.4 Brain MRI acquisition and post‐processing

Participants were scanned at the Vanderbilt University Institute of Imaging Science with a 3T Philips Achieva system. From 2012 to 2017, images were collected using an 8‐channel SENSE receiver head coil before upgrading to a 32‐channel dStream head coil with digital gradient coils and new software in 2017. Imaging parameters have been previously described.^18^ T1 images were post‐processed using Multi‐Atlas^19^ for volumes and FreeSurfer (http://surfer.nmr.mgh.harvard.edu/) for cortical thickness. For FreeSurfer segmentation, surfaces were manually inspected and corrected for registration, topological, and segmentation defects. After manual correction, images were reprocessed to update the transformation template and segmentation information. An AD signature region composite measure was calculated by summing bilateral cortical thickness measurements from regions shown to distinguish cognitively unimpaired individuals from those individuals with AD.^20^ Lower AD signature composite values indicate greater AD‐related cortical thinning and atrophy. Volumes of specific regions of interest were calculated, including frontal, parietal, temporal, and occipital lobe volume; hippocampal volume; and inferior lateral ventricle volume.

T2 fluid‐attenuated inversion recovery (FLAIR) images were post‐processed using the Lesion Segmentation Tool toolbox for Statistical Parametric Mapping (SPM8)^21^ to identify white matter hyperintensities (WMHs). Scans were reviewed and manually corrected for mislabeling. Manual corrections were then confirmed by a board‐certified neuroradiologist blinded to clinical information (L.T.D.) using the Medical Image Processing, Analysis, and Visualization application (https://mipav.cit.nih.gov). FLAIR variables of interest included frontal, parietal, temporal, and occipital lobe WMHs.

Prior to analysis, we applied the ComBat method to harmonize our longitudinally acquired neuroimaging data.^22^ Longitudinal ComBat input includes features to be harmonized, specification of batch variables, and specification of a linear mixed effects model. Advantages of this approach include controlling type I errors and enhancing the detection of the imaging features of interest.

### 2.5 Covariates

All covariates were collected at study entry and determined a priori based on their potential to confound analytical models, as previously described.^23^ Covariates included age, sex, race/ethnicity, education, apolipoprotein E (*APOE*) ε4 carrier status (defined as positive [ε2/ε4, ε3/ε4, ε4/ε4] or negative [ε2/ε2, ε2/ε3, ε3/ε3]), Framingham Stroke Risk Profile (FSRP) excluding points for age, and cognitive status.

### 2.6 Analytical plan

Prior to examining blood biomarker x kidney function interaction models, we first sought to replicate past findings in the field associating abnormal blood biomarkers with poorer brain health outcomes. We used linear regression models with ordinary least squares estimates, adjusting for covariates as listed above, to cross‐sectionally relate baseline plasma biomarkers (GFAP, NfL, Aβ42, and p‐tau) individually to baseline neuropsychological (Boston Naming Test [BNT], Animal Fluency, Delis‐Kaplan Executive Function System [D‐KEFS] Number Sequencing, Digit‐Symbol Coding, Hooper Visual Organization Test [HVOT], Executive Function Composite, Episodic Memory Composite), and neuroimaging (AD signature cortical thickness composite; frontal, temporal, parietal, and occipital lobe gray matter volume; hippocampal volume; inferior lateral ventricle volume; and frontal, temporal, parietal, and occipital lobe WMHs) outcomes. For longitudinal analyses, linear mixed‐effects models with follow‐up time interactions, adjusting for covariates listed above, related each baseline plasma biomarker individually to each cognitive and neuroimaging outcome over the follow‐up period.

To test the hypothesis that associations between blood‐based biomarkers and brain health outcomes would be weaker in the presence of reduced eGFR levels, follow‐up models assessed blood‐based biomarkers x eGFR interactions on baseline and longitudinal outcomes, with subsequent models stratified by CKD staging (stage 1/no CKD: eGFR > 90 mL/minute/1.73m^2^; stage 2: eGFR = 60–89 mL/minute/1.73m^2^; stage 3: eGFR = 30–59 mL/minute/1.73m^2^). No participants met criteria for stage 4 or 5.

To determine whether outliers influenced results, sensitivity analyses excluded predictor or outcome values > 4 standard deviations from the group mean. A multiple comparison correction was performed across outcomes per analytical set using a false discovery rate (FDR) based on the Benjamini–Hochberg procedure.^24^ Analyses were performed using R 4.2.2 (www.r‐project.org), and significance was set a priori at *p* < 0.05.

## 3 RESULTS

### 3.1 Participant characteristics

**FIGURE 1:**
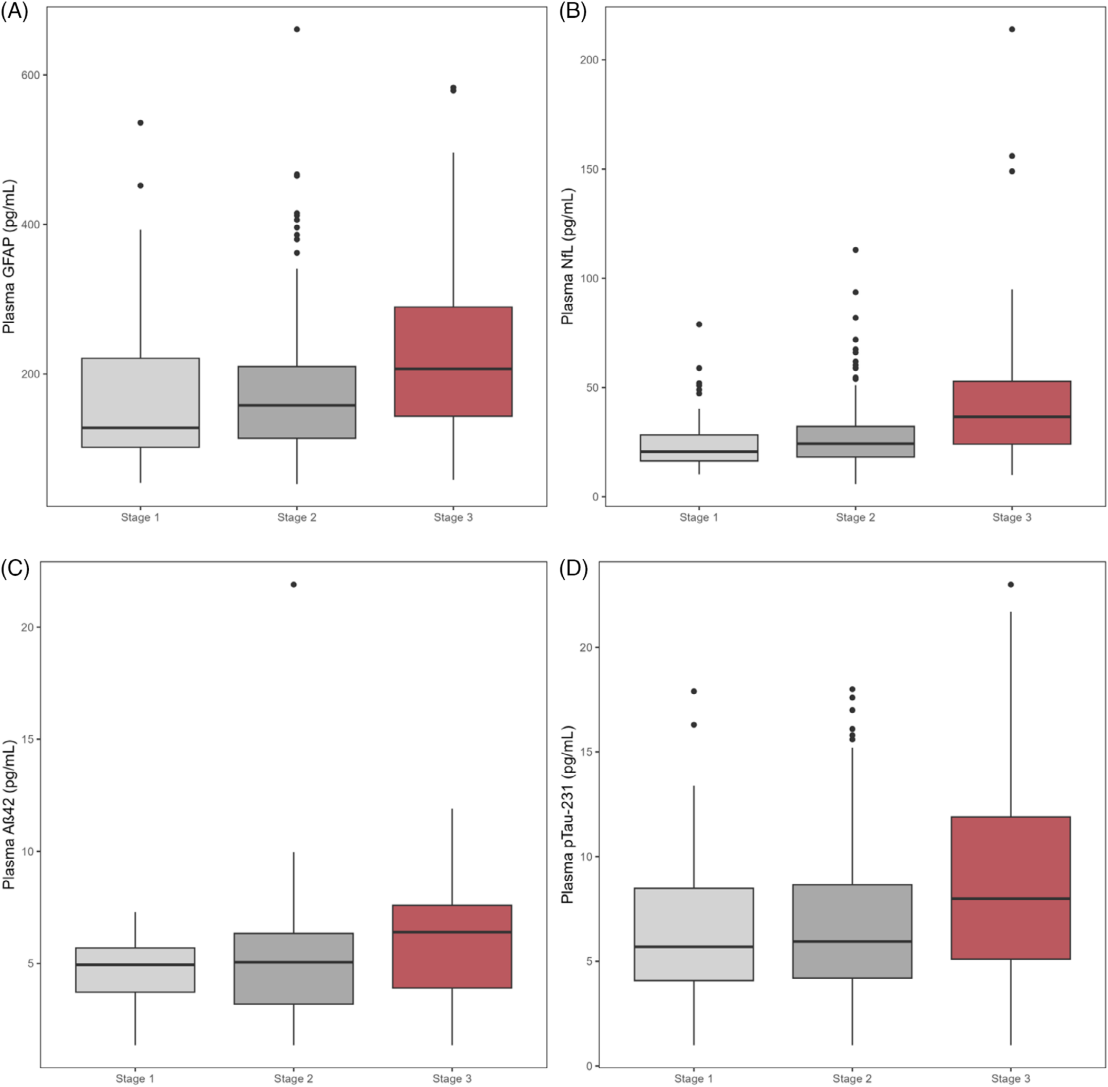
Plasma biomarker levels by CKD stage. CKD staging is based on eGFR (stage 1/no CKD: eGFR > 90 mL/minute/1.73m^2^; stage 2: eGFR = 60–89 mL/minute/1.73m^2^; stage 3: eGFR = 30–59 mL/minute/1.73m^2^). Boxes represent the interquartile range, with horizontal bars inside boxes representing the median biomarker value. Whiskers indicate minimum and maximum values (excluding outliers), and dots represent outliers > 4 SD from the mean. Aβ, amyloid beta; CKD, chronic kidney disease; eGFR, estimated glomerular filtration rate; GFAP, glial fibrillary acidic protein; NfL, neurofilament light chain; p‐tau, phosphorylated tau; SD, standard deviation.

Participants included 333 adults aged 60 to 92 at baseline (41% female, 35% *APOE* ε4 carriers, 16 ± 3 years of education). The sample was predominantly non‐Hispanic White (86%) or non‐Hispanic Black (11%). The mean eGFR for the entire sample was 74 ± 16 mL/minute/1.73m^2^ (range: 32–123) with 17% having no CKD or stage 1 CKD, 65% having stage 2 CKD, and 18% having stage 3 CKD. No participants had stage 4 (severe) or 5 (kidney failure) CKD. The mean follow‐up time was 6.4 ± 2.8 (range 1.4–9.7) years for neuropsychological outcomes and 6.0 ± 2.9 (range 0.8–9.6) years for neuroimaging outcomes. Significant between‐group differences across CKD stages were seen for age (*p* < 0.001), race/ethnicity (*p* = 0.03), FSRP score (*p* < 0.001), and all plasma biomarkers (*p* < 0.02). Stage 3 CKD was associated with higher plasma GFAP, NfL, Aβ42, and p‐tau_231_ levels (*p* values < 0.004) compared to no CKD/stage 1 and stage 2 CKD. Rates of CSF amyloid positivity (*p *= 0.20), *APOE* ε4 status (*p* = 0.62), and cognitive status at study entry (*p* = 0.73) were similar across groups. See Table 1 for participant characteristics for the combined sample as well as stratified by CKD stage and Table S1 in supporting information for annual change rates for each outcome stratified by CKD stage. See Figure 1 for an illustration of biomarker values by CKD stage.

### 3.2 Plasma biomarkers and cognition

In cross‐sectional models, plasma GFAP was associated with scores on the BNT, Animal Naming, executive function composite, and episodic memory composite (*p* values < 0.04). Plasma NfL was cross‐sectionally associated with scores on the BNT, Animal Naming, HVOT, executive function composite, and episodic memory composite (*p* values < 0.05). Plasma Aβ42 was not cross‐sectionally associated with any neuropsychological scores (*p* values < 0.10). Plasma p‐tau_231_ was cross‐sectionally associated with executive function and episodic memory composite scores (*p* values < 0.05). See Table S2 in supporting information for more detailed results of cross‐sectional main effect models.

In cross‐sectional interaction models, plasma NfL interacted with eGFR on Coding (*p* = 0.02) and HVOT scores (*p* = 0.04). Plasma Aβ42 interacted with eGFR on episodic memory composite scores (*p* = 0.03). In stratified models, associations between biomarkers and cognitive outcomes were stronger in individuals with better kidney function than in individuals with worse kidney function. All significant interactions were attenuated after correction for multiple comparisons (*p* values_FDR _> 0.24). Other plasma biomarkers did not interact with eGFR on any cross‐sectional neuropsychological outcomes (*p* values > 0.05). See Table S3 in supporting information for results of cross‐sectional interaction and stratified models.

In longitudinal models, plasma GFAP, NfL, and p‐tau_231_ were each associated with trajectories of all cognitive outcomes (*p* values < 0.004). Plasma Aβ42 was not associated with any neuropsychological outcome (*p* values > 0.05). See Table S4 in supporting information for detailed results of longitudinal main effect models.

**FIGURE 2:**
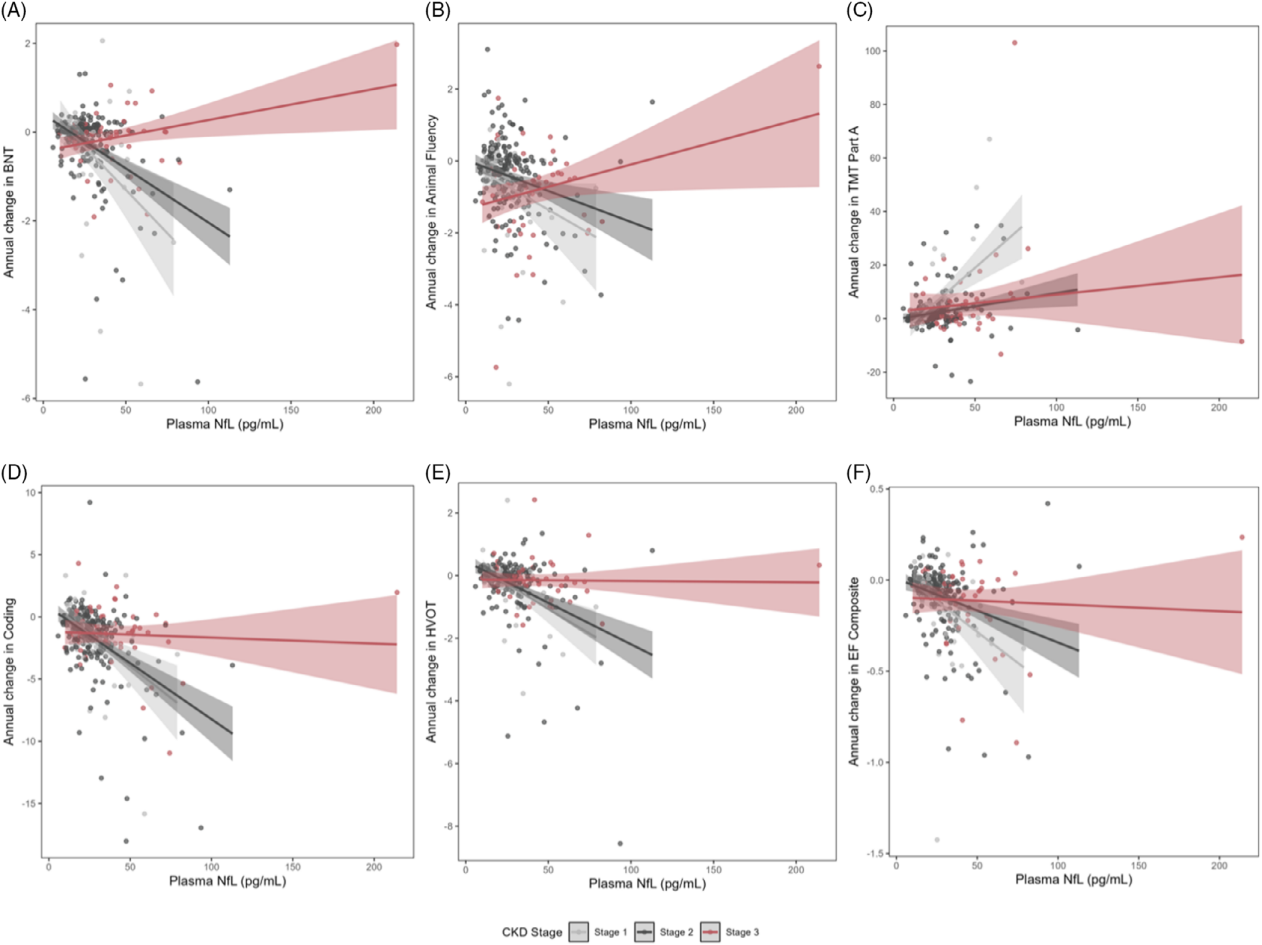
Longitudinal plasma NfL x eGFR interactions on neuropsychological outcomes. Lines reflect an annual change in neuropsychological outcomes corresponding to plasma NfL level, stratified by CKD stage. Shading reflects a 95% confidence interval. A, Associations between plasma NfL and annual change in BNT, stage 1/no CKD: β = –0.05, *p *< 0.0001; stage 2: β = –0.02, *p* < 0.0001; stage 3: β = 0.002, *p* = 0.57. B, Associations between plasma NfL and annual change in Animal Fluency, stage 1/no CKD: β = –0.05, *p* = 0.0003; stage 2: β = –0.02, *p* = 0.001; stage 3: β = 0.003, *p* = 0.69. C, Associations between plasma NfL and annual change in Number Sequencing, stage 1/no CKD: β = 0.50, *p* < 0.0001; stage 2: β = 0.06, *p* = 0.03; stage 3: β = 0.11, *p* = 0.002. D, Associations between plasma NfL and annual change in Coding, stage 1/no CKD: β = –0.06, *p* = 0.001; stage 2: β = –0.06, *p* < 0.0001; stage 3: β = –0.02, *p* = 0.15. E, Associations between plasma NfL and annual change in HVOT, stage 1/no CKD: β = –0.03, *p* < 0.0001; stage 2: β = –0.02, *p* < 0.0001; stage 3: β = –0.008, *p* = 0.02. F, Associations between plasma NfL and annual change in EF Composite, stage 1/no CKD: β = –0.007, *p* < 0.0001; stage 2: β = –0.004, *p* < 0.0001; stage 3: β = –0.003, *p* = 0.002. BNT, Boston Naming Test; CKD, chronic kidney disease; EF, executive function; eGFR, estimated glomerular filtration rate; HVOT, Hooper Visual Organization Test; NfL, neurofilament light chain; TMT, Trail‐Making Test.

In longitudinal interaction models, plasma GFAP interacted with eGFR on longitudinal BNT scores (*p* = 0.02). Plasma NfL interacted with eGFR on longitudinal trajectories of BNT, Animal Naming, Number Sequencing, Coding, HVOT, and executive composite (*p* values < 0.03; see Figure 2). Plasma Aβ42 interacted with eGFR on longitudinal BNT (*p* = 0.02) and Animal Naming (*p* = 0.01) scores. Plasma p‐tau_231_ interacted with eGFR on longitudinal executive function composite score (*p* = 0.04). In stratified models, associations between plasma biomarkers and longitudinal cognitive outcomes were generally stronger in individuals with better kidney function. Interactions between plasma NfL and eGFR on BNT, Animal Naming, Number Sequencing, HVOT, and executive function composite trajectories persisted after correction for multiple comparisons and were largely unchanged in sensitivity analyses excluding outliers. All other interactions were attenuated after correction for multiple comparisons (*p* values_FDR _> 0.09). See Table 2 for the results of longitudinal interaction and stratified models.

**TABLE 2:** Blood‐based biomarker x eGFR interactions on longitudinal clinical outcomes.

	Interaction Model (*n* = 333)			No CKD/Stage 1 (*n* = 57)			Stage 2 CKD (*n* = 217)			Stage 3 CKD (*n* = 59)		
	β	95% CI	*p*	β	95% CI	*p*	β	95% CI	*p*	β	95% CI	*p*
**Plasma GFAP**												
BNT	−5.8E‐5	−1.1E‐4, −8.6E‐6	**0.02**	−3.7E‐3	−6.5E‐3, −9.9E‐4	***0.01***	−3.4E‐3	−4.4E‐3, −2.4E‐3	***<0.0001***	−8.5E‐4	−2.0E‐3, 3.3E‐4	0.16
Animal Fluency	−1.6E‐5	−7.6E‐5, 4.3E‐5	0.59	−3.0E‐3	−5.6E‐3, −3.6E‐4	**0.03**	−1.9E‐3	−3.2E‐3, −6.0E‐4	***0.004***	−4.4E‐4	−2.7E‐3, 1.8E‐3	0.70
Number Sequencing	−2.8E‐4	−7.2E‐4, 1.6E‐4	0.21	1.8E‐2	−1.5E‐2, 5.1E‐2	0.28	1.3E‐2	5.1E‐3, 2.0E‐2	***0.001***	1.7E‐2	3.8E‐3, 3.0E‐2	**0.01**
Coding	−3.0E‐5	−1.5E‐4, 9.2E‐5	0.63	−7.1E‐3	−1.2E‐2, −2.7E‐3	***0.002***	−7.5E‐3	−1.0E‐2, −4.7E‐3	***<0.0001***	−2.9E‐3	−7.1E‐3, 1.2E‐3	0.17
HVOT	−4.1E‐5	−8.3E‐5, 1.9E‐6	0.06	−3.1E‐3	−5.0E‐3, −1.2E‐3	***0.002***	−3.1E‐3	−4.0E‐3, −2.2E‐3	***<0.0001***	−1.1E‐3	−2.4E‐3, 2.3E‐4	0.10
EF Composite	−1.4E‐6	−1.0E‐5, 7.5E‐6	0.76	−4.6E‐4	−8.2E‐4, −1.1E‐4	***0.01***	−4.3E‐4	−6.2E‐4, −2.4E‐4	***<0.0001***	−2.8E‐4	−6.8E‐4, 1.3E‐4	0.18
Memory Composite	4.4E‐6	−4.0E‐6, 1.3E‐5	0.30	−4.5E‐4	−7.3E‐4, −1.8E‐4	***0.001***	−3.5E‐4	−5.3E‐4, −1.6E‐4	***0.0003***	−5.7E‐4	−8.9E‐4, −2.5E‐4	***0.001***
AD signature	−1.6E‐7	−1.3E‐6, 1.0E‐6	0.79	−2.8E‐5	−7.6E‐5, 2.1E‐5	0.26	−1.2E‐5	−3.5E‐5, 1.2E‐5	0.34	−1.2E‐5	−6.1E‐5, 3.6E‐5	0.61
Frontal lobe GM	1.8E‐2	−2.1E‐1, 2.5E‐1	0.88	−4.1E+0	−1.2E+1, 3.5E+0	0.28	−6.6E+0	−1.2E+1, −1.2E+0	**0.02**	−1.4E+0	−1.3E+1, 9.9E+0	0.81
Temporal lobe GM	−4.4E‐3	−8.3E‐2, 7.5E‐2	0.91	−3.0E+0	−5.3E+0, −6.8E‐1	**0.01**	−3.3E+0	−5.0E+0, −1.5E+0	***0.0002***	−2.4E+0	−5.8E+0, 9.9E‐1	0.16
Parietal lobe GM	9.1E‐3	−1.1E‐1, 1.3E‐1	0.88	−3.4E+0	−7.2E+0, 3.9E‐1	0.08	−4.2E+0	−6.8E+0, −1.6E+0	***0.001***	−2.2E+0	−7.8E+0, 3.4E+0	0.44
Occipital lobe GM	−3.0E‐2	−8.1E‐2, 2.0E‐2	0.24	−2.7E+0	−4.9E+0, −4.9E‐1	**0.02**	−1.7E+0	−2.7E+0, −6.8E‐1	***0.001***	−4.5E‐1	−2.7E+0, 1.8E+0	0.69
Hippocampus	1.2E‐3	−4.6E‐3, 7.0E‐3	0.68	−2.5E‐1	−4.6E‐1, −3.4E‐2	**0.02**	−3.4E‐1	−4.7E‐1, −2.1E‐1	***<0.0001***	−2.1E‐1	−4.1E‐1, −8.5E‐3	**0.04**
ILV	7.6E‐4	−9.8E‐3, 1.1E‐2	0.89	9.4E‐1	3.5E‐1, 1.5E+0	***0.002***	7.2E‐1	4.7E‐1, 9.8E‐1	***<0.0001***	7.1E‐1	2.6E‐1, 1.2E+0	**0.002**
Frontal WMH	9.3E‐5	2.3E‐5, 1.6E‐4	**0.01**	6.2E‐3	1.6E‐3, 1.1E‐2	***0.01***	2.9E‐3	1.3E‐3, 4.4E‐3	***0.0003***	8.0E‐4	−1.8E‐3, 3.4E‐3	0.54
Temporal WMH	4.5E‐6	−7.9E‐6, 1.7E‐5	0.48	5.6E‐4	−2.8E‐4, 1.4E‐3	0.19	9.1E‐5	−1.3E‐4, 3.1E‐4	0.41	5.2E‐4	−2.1E‐4, 1.2E‐3	0.16
Parietal WMH	3.0E‐5	−1.1E‐5, 7.1E‐5	0.15	3.4E‐3	9.7E‐4, 5.8E‐3	***0.01***	1.2E‐3	2.1E‐4, 2.2E‐3	**0.02**	1.1E‐3	−4.1E‐4, 2.6E‐3	0.15
Occipital WMH	2.6E‐5	9.6E‐7, 5.1E‐5	**0.04**	1.7E‐3	8.4E‐4, 2.6E‐3	***0.0002***	4.1E‐4	−1.4E‐4, 9.7E‐4	0.14	3.0E‐5	−9.5E‐4, 1.0E‐3	0.95
**Plasma NfL**												
BNT	−9.6E‐4	−1.3E‐3, −6.5E‐4	***<0.0001***	−4.6E‐2	−6.4E‐2, −2.9E‐2	***<0.0001***	−2.1E‐2	−2.8E‐2, −1.4E‐2	***<0.0001***	1.8E‐3	−4.6E‐3, 8.3E‐3	0.57
Animal Fluency	−6.8E‐4	−1.1E‐3, −2.9E‐4	***0.001***	−3.3E‐2	−5.1E‐2, −1.5E‐2	***0.0003***	−1.7E‐2	−2.6E‐2, −7.0E‐3	***0.001***	−2.6E‐3	−1.5E‐2, 1.0E‐2	0.69
Number Sequencing	4.5E‐3	1.6E‐3, 7.4E‐3	***0.002***	5.0E‐1	2.8E‐1, 7.2E‐1	***<0.0001***	6.4E‐2	6.8E‐3, 1.2E‐1	**0.03**	1.1E‐1	3.8E‐2, 1.7E‐1	***0.002***
Coding	−1.0E‐3	−1.9E‐3, −1.7E‐4	**0.02**	−5.9E‐2	−9.5E‐2, −2.4E‐2	***0.001***	−6.0E‐2	−8.1E‐2, −4.0E‐2	***<0.0001***	−1.6E‐2	−3.9E‐2, 6.3E‐3	0.15
HVOT	−6.7E‐4	−9.4E‐4, −4.1E‐4	***<0.0001***	−3.4E‐2	−4.7E‐2, −2.2E‐2	***<0.0001***	−2.0E‐2	−2.6E‐2, −1.3E‐2	***<0.0001***	−8.4E‐3	−1.6E‐2, −1.2E‐3	**0.02**
EF composite	−1.0E‐4	−1.6E‐4, −4.7E‐5	***0.0003***	−6.5E‐3	−8.6E‐3, −4.3E‐3	***<0.0001***	−3.9E‐3	−5.3E‐3, −2.5E‐3	***<0.0001***	−3.0E‐3	−5.0E‐3, −1.1E‐3	***0.002***
Memory composite	−2.5E‐5	−8.1E‐5, 3.0E‐5	0.37	−3.4E‐3	−5.5E‐3, −1.4E‐3	***0.001***	−2.9E‐3	−4.2E‐3, −1.5E‐3	***<0.0001***	−2.4E‐3	−4.2E‐3, −5.8E‐4	**0.01**
AD signature	−2.3E‐6	−1.2E‐5, 7.1E‐6	0.63	−3.7E‐4	−7.7E‐4, 3.7E‐5	0.07	−1.6E‐5	−2.1E‐4, 1.8E‐4	0.87	−2.2E‐4	−5.1E‐4, 5.8E‐5	0.12
Frontal lobe GM	8.7E‐1	−7.7E‐1, 2.5E+0	0.30	−1.5E+1	−7.6E+1, 4.5E+1	0.61	−2.6E+1	−6.7E+1, 1.5E+1	0.21	−2.0E+1	−7.7E+1, 3.6E+1	0.48
Temporal lobe GM	−1.0E‐1	−6.5E‐1, 4.5E‐1	0.72	−2.8E+1	−4.5E+1, −1.0E+1	***0.002***	−1.5E+1	−2.9E+1, −2.1E+0	**0.02**	−2.0E+1	−3.7E+1, −3.2E+0	**0.02**
Parietal lobe GM	2.5E‐1	−5.7E‐1, 1.1E+0	0.55	−2.4E+1	−5.4E+1, 5.5E+0	0.11	−2.3E+1	−4.3E+1, −3.0E+0	**0.02**	−1.9E+1	−4.7E+1, 8.8E+0	0.18
Occipital lobe GM	−2.4E‐1	−5.9E‐1, 1.2E‐1	0.19	−2.4E+1	−4.0E+1, −7.9E+0	***0.004***	−8.2E+0	−1.6E+1, −5.0E‐1	**0.04**	−7.4E+0	−1.9E+1, 3.8E+0	0.19
Hippocampus	−5.2E‐2	−9.0E‐2, −1.4E‐2	**0.01**	−2.9E+0	−4.3E+0, −1.4E+0	***0.0002***	−2.1E+0	−3.1E+0, −1.2E+0	***<0.0001***	−5.0E‐1	−1.5E+0, 4.8E‐1	0.32
ILV	1.1E‐1	4.0E‐2, 1.7E‐1	***0.002***	1.1E+1	6.7E+0, 1.4E+1	***<0.0001***	5.5E+0	3.9E+0, 7.1E+0	***<0.0001***	1.6E+0	−2.4E‐1, 3.5E+0	0.09
Frontal WMH	9.6E‐4	5.3E‐4, 1.4E‐3	***<0.0001***	5.4E‐2	2.0E‐2, 8.8E‐2	***0.002***	1.7E‐2	6.3E‐3, 2.7E‐2	***0.002***	1.9E‐3	−1.1E‐2, 1.5E‐2	0.78
Temporal WMH	−2.3E‐5	−1.1E‐4, 6.3E‐5	0.60	3.8E‐3	−2.7E‐3, 1.0E‐2	0.25	8.4E‐4	−7.9E‐4, 2.5E‐3	0.31	1.0E‐3	−3.0E‐3, 5.0E‐3	0.62
Parietal WMH	3.3E‐4	6.6E‐5, 5.9E‐4	**0.01**	2.7E‐2	8.5E‐3, 4.5E‐2	***0.004***	5.7E‐3	−8.5E‐4, 1.2E‐2	0.09	6.3E‐3	−6.0E‐4, 1.3E‐2	0.07
Occipital WMH	1.2E‐4	−5.0E‐5, 2.9E‐4	0.17	1.1E‐2	4.7E‐3, 1.8E‐2	***0.001***	1.5E‐3	−2.6E‐3, 5.7E‐3	0.47	2.6E‐3	−2.9E‐3, 8.1E‐3	0.35
**Plasma Aβ42**												
BNT	3.2E‐3	6.2E‐4, 5.8E‐3	**0.02**	1.4E‐1	−5.0E‐2, 3.3E‐1	0.15	5.8E‐3	−3.0E‐2, 4.2E‐2	0.75	−5.7E‐2	−1.1E‐1, −6.9E‐3	**0.03**
Animal Fluency	3.3E‐3	7.1E‐4, 5.9E‐3	**0.01**	1.0E‐1	−4.7E‐2, 2.5E‐1	0.18	2.8E‐2	−1.4E‐2, 7.0E‐2	0.19	−5.9E‐2	−1.5E‐1, 2.7E‐2	0.18
Number Sequencing	−9.6E‐3	−3.0E‐2, 1.1E‐2	0.35	−4.4E‐1	−2.4E+0, 1.5E+0	0.65	1.2E‐1	−1.3E‐1, 3.6E‐1	0.34	2.4E‐1	−2.7E‐1, 7.5E‐1	0.35
Coding	4.7E‐3	−6.0E‐4, 1.0E‐2	0.08	5.2E‐2	−1.8E‐1, 2.8E‐1	0.66	1.2E‐2	−8.2E‐2, 1.1E‐1	0.80	−1.0E‐1	−2.6E‐1, 5.7E‐2	0.21
HVOT	9.6E‐4	−1.1E‐3, 3.0E‐3	0.36	1.0E‐1	−2.4E‐2, 2.3E‐1	0.11	1.6E‐2	−1.5E‐2, 4.7E‐2	0.32	1.3E‐2	−4.3E‐2, 6.9E‐2	0.64
EF composite	3.7E‐5	−3.8E‐4, 4.5E‐4	0.86	8.2E‐3	−1.3E‐2, 3.0E‐2	0.45	−1.3E‐4	−6.5E‐3, 6.2E‐3	0.97	5.9E‐3	−1.1E‐2, 2.3E‐2	0.51
Memory composite	2.6E‐4	−1.3E‐4, 6.4E‐4	0.20	7.2E‐3	−1.0E‐2, 2.4E‐2	0.41	1.4E‐3	−4.8E‐3, 7.6E‐3	0.66	3.8E‐3	−1.1E‐2, 1.9E‐2	0.62
AD signature	2.2E‐5	−2.7E‐5, 7.1E‐5	0.38	1.3E‐4	−2.3E‐3, 2.5E‐3	0.92	1.9E‐4	−5.3E‐4, 9.0E‐4	0.60	−7.5E‐4	−2.6E‐3, 1.1E‐3	0.42
Frontal lobe GM	5.9E+0	−3.9E+0, 1.6E+1	0.24	1.1E+2	−3.0E+2, 5.1E+2	0.61	−1.4E+2	−3.2E+2, 3.7E+1	0.12	−1.3E+2	−5.7E+2, 3.0E+2	0.54
Temporal lobe GM	2.0E+0	−1.4E+0, 5.3E+0	0.25	7.1E+1	−6.0E+1, 2.0E+2	0.29	−1.6E+1	−7.2E+1, 4.1E+1	0.59	−1.9E+1	−1.5E+2, 1.1E+2	0.77
Parietal lobe GM	3.8E+0	−1.2E+0, 8.8E+0	0.13	9.6E+1	−1.1E+2, 3.0E+2	0.35	−7.1E+1	−1.6E+2, 1.4E+1	0.10	−9.9E+1	−3.1E+2, 1.1E+2	0.36
Occipital lobe GM	3.6E‐1	−1.9E+0, 2.6E+0	0.76	6.1E+1	−7.9E+1, 2.0E+2	0.39	−3.4E+1	−6.6E+1, −1.1E+0	**0.04**	−1.4E+1	−1.0E+2, 7.6E+1	0.76
Hippocampus	2.0E‐1	−8.7E‐2, 4.8E‐1	0.18	9.4E+0	−5.5E+0, 2.4E+1	0.21	−6.5E‐1	−5.0E+0, 3.7E+0	0.77	2.8E‐1	−8.4E+0, 9.0E+0	0.95
ILV	−4.1E‐2	−5.7E‐1, 4.9E‐1	0.88	−3.2E+1	−7.5E+1, 1.2E+1	0.15	−3.3E+0	−1.2E+1, 5.5E+0	0.46	4.2E+0	−1.7E+1, 2.5E+1	0.70
Frontal WMH	−9.7E‐4	−4.4E‐3, 2.5E‐3	0.58	−3.8E‐1	−7.0E‐1, −7.1E‐2	**0.02**	5.0E‐2	−1.4E‐3, 1.0E‐1	0.06	−5.2E‐2	−1.6E‐1, 6.0E‐2	0.36
Temporal WMH	−2.1E‐4	−7.4E‐4, 3.3E‐4	0.45	−4.1E‐2	−9.3E‐2, 1.0E‐2	0.12	5.3E‐3	−1.7E‐3, 1.2E‐2	0.14	6.5E‐3	−2.4E‐2, 3.7E‐2	0.67
Parietal WMH	−1.3E‐3	−3.2E‐3, 6.3E‐4	0.19	−2.1E‐1	−3.7E‐1, −4.5E‐2	**0.01**	3.4E‐2	1.9E‐3, 6.7E‐2	**0.04**	−2.3E‐2	−8.5E‐2, 4.0E‐2	0.48
Occipital WMH	−9.3E‐4	−2.0E‐3, 1.6E‐4	0.10	−6.7E‐2	−1.2E‐1, −1.6E‐2	**0.01**	7.6E‐3	−1.0E‐2, 2.5E‐2	0.40	2.4E‐2	−1.2E‐2, 6.0E‐2	0.20
**Plasma p‐tau_231_**												
BNT	−8.9E‐4	−2.0E‐3, 2.6E‐4	0.13	−8.0E‐2	−1.6E‐1, −5.0E‐3	**0.04**	−4.6E‐2	−7.0E‐2, −2.2E‐2	***0.0002***	−4.1E‐2	−6.6E‐2, −1.6E‐2	**0.002**
Animal Fluency	−5.0E‐4	−1.7E‐3, 7.5E‐4	0.44	−4.2E‐2	−1.1E‐1, 2.0E‐2	0.18	−4.5E‐2	−7.3E‐2, −1.8E‐2	***0.001***	−1.1E‐2	−5.9E‐2, 3.8E‐2	0.67
Number Sequencing	2.9E‐3	−6.4E‐3, 1.2E‐2	0.54	3.9E‐1	−4.3E‐1, 1.2E+0	0.35	3.7E‐1	2.1E‐1, 5.3E‐1	***<0.0001***	2.3E‐1	−5.6E‐2, 5.1E‐1	0.12
Coding	−5.3E‐4	−3.1E‐3, 2.1E‐3	0.69	−1.1E‐1	−2.2E‐1, −9.9E‐3	**0.03**	−5.3E‐2	−1.2E‐1, 1.1E‐2	0.10	−1.1E‐1	−2.0E‐1, −2.9E‐2	**0.01**
HVOT	−5.2E‐5	−1.8E‐4, 7.2E‐5	0.41	8.1E‐4	−7.0E‐3, 8.6E‐3	0.84	−1.7E‐4	−2.3E‐3, 2.0E‐3	0.88	7.5E‐4	−2.5E‐3, 4.0E‐3	0.65
EF composite	−2.1E‐4	−4.0E‐4, −1.2E‐5	**0.04**	−8.7E‐3	−1.7E‐2, 1.9E‐4	0.06	−6.3E‐3	−1.1E‐2, −2.1E‐3	***0.004***	1.8E‐3	−7.2E‐3, 1.1E‐2	0.70
Memory composite	9.5E‐5	−8.6E‐5, 2.8E‐4	0.30	−5.4E‐3	−1.3E‐2, 1.8E‐3	0.14	−5.1E‐3	−9.2E‐3, −9.9E‐4	**0.02**	−8.7E‐3	−1.6E‐2, −1.4E‐3	**0.02**
AD signature	−1.0E‐5	−3.4E‐5, 1.4E‐5	0.40	−4.2E‐4	−1.5E‐3, 6.3E‐4	0.43	−1.9E‐4	−7.0E‐4, 3.2E‐4	0.46	−2.3E‐4	−1.2E‐3, 7.7E‐4	0.65
Frontal lobe GM	4.9E+0	−2.3E‐2, 9.8E+0	0.05	7.3E+1	−1.0E+2, 2.5E+2	0.41	−6.7E+0	−1.3E+2, 1.1E+2	0.91	−1.4E+2	−3.8E+2, 9.4E+1	0.24
Temporal lobe GM	1.9E‐1	−1.5E+0, 1.9E+0	0.83	−2.0E+1	−7.6E+1, 3.5E+1	0.47	−1.6E+1	−5.5E+1, 2.3E+1	0.42	−2.3E+1	−9.4E+1, 4.8E+1	0.53
Parietal lobe GM	2.0E+0	−4.5E‐1, 4.5E+0	0.11	2.6E+1	−6.2E+1, 1.1E+2	0.56	−1.3E+1	−7.2E+1, 4.5E+1	0.66	−6.4E+1	−1.8E+2, 5.3E+1	0.28
Occipital lobe GM	−4.5E‐1	−1.6E+0, 6.6E‐1	0.43	−3.2E+1	−8.7E+1, 2.2E+1	0.25	1.5E+0	−2.1E+1, 2.4E+1	0.90	−1.4E+1	−6.2E+1, 3.3E+1	0.55
Hippocampus	3.7E‐2	−9.1E‐2, 1.6E‐1	0.57	2.6E‐1	−5.5E+0, 6.0E+0	0.93	−5.3E+0	−8.2E+0, −2.4E+0	***0.0004***	−3.2E+0	−7.6E+0, 1.2E+0	0.16
ILV	9.2E‐2	−1.4E‐1, 3.2E‐1	0.44	1.3E+1	−3.4E+0, 3.0E+1	0.12	1.1E+1	4.7E+0, 1.7E+1	***0.0004***	9.8E+0	−7.3E‐1, 2.0E+1	0.07
Frontal WMH	1.5E‐3	−7.7E‐5, 3.0E‐3	0.06	4.1E‐2	−8.9E‐2, 1.7E‐1	0.53	4.9E‐2	1.4E‐2, 8.4E‐2	***0.01***	2.3E‐2	−3.5E‐2, 8.1E‐2	0.43
Temporal WMH	−1.4E‐4	−4.1E‐4, 1.2E‐4	0.29	−4.1E‐3	−2.6E‐2, 1.8E‐2	0.72	8.0E‐3	3.2E‐3, 1.3E‐2	***0.001***	1.9E‐2	3.4E‐3, 3.5E‐2	**0.02**
Parietal WMH	6.4E‐4	−2.5E‐4, 1.5E‐3	0.16	3.1E‐2	−3.7E‐2, 9.9E‐2	0.37	2.8E‐2	6.1E‐3, 5.0E‐2	**0.01**	1.8E‐2	−1.5E‐2, 5.0E‐2	0.28
Occipital WMH	7.5E‐5	−4.6E‐4, 6.1E‐4	0.78	2.6E‐2	1.6E‐3, 5.1E‐2	**0.04**	1.3E‐2	4.1E‐4, 2.5E‐2	**0.04**	3.0E‐2	1.0E‐2, 5.0E‐2	**0.003**

*Note*: Models were adjusted for age, sex, education, race/ethnicity, *APOE* ε4 status, Framingham Stroke Risk Profile, and cognitive status. β Indicates the degree of change in outcomes per 1 unit increase in the respective blood‐based biomarker. Bold font indicates *p* < 0.05. Italic font indicates significant associations persisted after FDR correction.

Abbreviations: Aβ, amyloid beta; AD, Alzheimer's disease; *APOE*, apolipoprotein E; BNT, Boston Naming Test; CI, confidence interval; CKD, chronic kidney disease; EF, executive function; eGFR, estimated glomerular filtration rate; FDR, false discovery rate; GFAP, glial fibrillary acidic protein; GM, gray matter; HVOT, Hooper Visual Organization Test; ILV, inferior lateral ventricle; MoCA, Montreal Cognitive Assessment; NfL, neurofilament light chain; p‐tau_,_ phosphorylated tau; WMH, white matter hyperintensity.

### 3.3 Plasma biomarkers and neuroimaging outcomes

In cross‐sectional models, plasma GFAP was associated with inferior lateral ventricle volume (*p* = 0.0007) and AD signature composite cortical thickness (*p* = 0.0006). Plasma NfL was cross‐sectionally associated with volumetric measures of the occipital lobe, the hippocampus, and the inferior lateral ventricle (*p* values < 0.05). Plasma Aβ42 was cross‐sectionally associated with frontal lobe volume (*p* = 0.01), parietal lobe volume (*p* = 0.02), and AD signature composite cortical thickness (*p* = 0.05). Plasma p‐tau_231_ was not associated with any cross‐sectional neuroimaging outcome (*p* values > 0.17). See Table S2 for detailed results of cross‐sectional main effect models.

In cross‐sectional interaction models, plasma GFAP interacted with eGFR on occipital lobe WMHs (*p* = 0.05) and AD signature composite (*p* = 0.05). Plasma NfL cross‐sectionally interacted with eGFR on inferior lateral ventricle volume (*p* = 0.0003) and temporal lobe WMHs (*p* = 0.03). Plasma Aβ42 and p‐tau_231_ did not interact with eGFR on any cross‐sectional neuroimaging outcome. In stratified models, associations between biomarkers and neuroimaging outcomes were generally stronger in individuals with better kidney function compared to those with worse kidney function. Interactions between plasma NfL and eGFR on inferior lateral ventricle volume persisted after correction for multiple comparisons and were largely unchanged in sensitivity analyses excluding outliers. All other significant interactions were attenuated after correction for multiple comparisons (*p* values_FDR _> 0.78). See Table S3 for the results of cross‐sectional interaction and stratified models.

In longitudinal models, plasma GFAP and NfL were each associated with frontal (GFAP *p* = 0.001; NfL *p* = 0.05), temporal (GFAP *p* < 0.0001; NfL *p *< 0.0001), parietal (GFAP *p *= 0.0008; NfL *p* = 0.003), and occipital lobe volumes (GFAP *p* = 0.0001; NfL *p* = 0.0001); hippocampal volume (GFAP *p* < 0.0001; NfL *p* < 0.0001); inferior lateral ventricle volume (GFAP *p* < 0.0001; NfL *p* < 0.0001); and frontal (GFAP *p* < 0.0001; NfL *p* < 0.0001), parietal (GFAP *p* = 0.0001; NfL *p* = 0.002), and occipital WMHs (GFAP *p* = 0.003; NfL *p* = 0.009). Plasma Aβ42 was associated with the longitudinal trajectory of parietal lobe gray matter volume (*p* = 0.03). Plasma p‐tau_231_ was associated with the longitudinal trajectory of hippocampal (*p* = 0.0002) and inferior lateral ventricle (*p* < 0.0001) volumes as well as frontal (*p* = 0.006), temporal (*p* = 0.004), parietal (*p *= 0.004), and occipital (*p* < 0.0001) WMHs. See Table S4 for detailed results of longitudinal main effect models.

**FIGURE 3:**
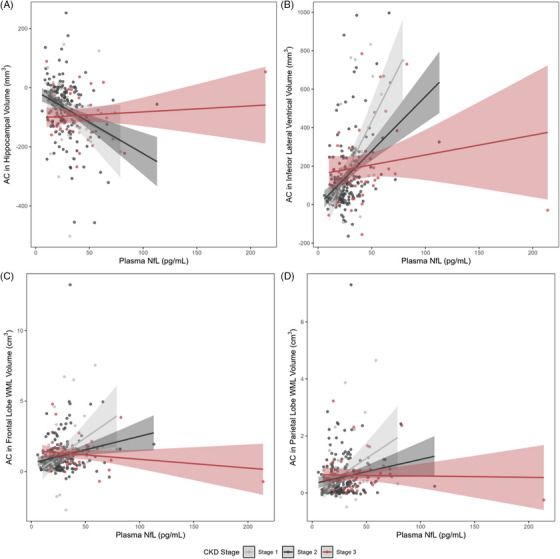
Longitudinal plasma NfL x eGFR interactions on neuroimaging outcomes. Lines reflect an annual change in neuroimaging outcomes corresponding to plasma NfL level, stratified by CKD stage. Shading reflects a 95% confidence interval. A, Associations between plasma NfL and annual change in hippocampal volume, stage 1/no CKD: β = –2.9, *p* = 0.0002; stage 2: β = –2.1, *p* < 0.0001; stage 3: β = –0.05, *p* = 0.32. B, Associations between plasma NfL and annual change in inferior lateral ventricle volume, stage 1/no CKD: β = 11.0, *p* < 0.0001; stage 2: β = 5.5, *p* < 0.0001; stage 3: β = 1.6, *p* = 0.09. C, Associations between plasma NfL and annual change in frontal lobe WMH volume, stage 1/no CKD: β = 0.05, *p* = 0.002; stage 2: β = 0.02, *p* = 0.002; stage 3: β = 0.002, *p* = 0.78. D, Associations between plasma NfL and annual change in parietal lobe WMH volume, stage 1/no CKD: β = 0.03, *p* = 0.004; stage 2: β = 0.006, *p* = 0.09; stage 3: β = 0.006, *p* = 0.07. AC, annual change; CKD, chronic kidney disease; eGFR, estimated glomerular filtration rate; NfL, neurofilament light chain; WMH, white matter hyperintensity; WML white matter lesion.

In longitudinal interaction models, plasma GFAP interacted with eGFR on longitudinal frontal (*p*  = 0.009) and occipital lobe WMHs (*p*  = 0.04). Plasma NfL interacted with eGFR on longitudinal hippocampal volume (*p* = 0.007), inferior lateral ventricle volume (*p* = 0.002), frontal WMHs (*p* < 0.0001), and parietal WMHs (*p* = 0.02; see Figure 3). Plasma Aβ42 and p‐tau_231_ did not interact with eGFR on any longitudinal neuroimaging outcome (*p* values > 0.05). In stratified models, associations between plasma biomarkers and longitudinal neuroimaging outcomes were generally stronger in individuals with better kidney function than in those with worse kidney function. Interactions between plasma NfL and eGFR on inferior lateral ventricle volume and frontal WMH trajectories persisted after correction for multiple comparisons and were largely unchanged in sensitivity analyses excluding outliers. All other interactions were attenuated after correction for multiple comparisons (*p* values_FDR _> 0.11). See Table 2 for the results of longitudinal interaction and stratified models.

## 4 DISCUSSION

In this community‐dwelling cohort of older adults without dementia or severe (stage 4 or 5) CKD, we found that even modest changes in kidney function impacted the predictive accuracy of plasma biomarkers of AD and concomitant pathologies for neuropsychological and neuroimaging outcomes. Plasma levels of GFAP, NfL, Aβ42, and p‐tau_231_ significantly differed across individuals grouped by eGFR‐based CKD stage, such that as kidney function decreased, plasma biomarker levels increased despite statistically adjusting for potential confounders like age, race/ethnicity, and cognitive status. While plasma p‐tau_231_, GFAP, and NfL were robust predictors of clinical (and especially longitudinal) outcomes, the predictive accuracy of these biomarkers was modified in many cases by higher eGFR levels such that associations were stronger in individuals with better kidney function (stage 0–2 CKD) and no longer present in individuals with stage 3 CKD. Taken together, results are consistent with past work highlighting the importance of considering kidney function when interpreting blood biomarkers, and expand this literature by showing that even mild kidney dysfunction can have significant impacts on the predictive accuracy of blood biomarkers for neuropsychological and neuroimaging outcomes.

The finding of increased levels of all plasma biomarkers in individuals with stage 3 CKD is notable. As participants across all CKD stages studied (stage 0–3) were statistically comparable for AD risk factors, such as amyloid positivity, cognitive status, and *APOE* ε4 carrier status, differences observed in plasma biomarkers of AD and concomitant pathologies do not appear to be due to group differences in AD risk or pathology. Indeed, while CKD is associated with increased incidence and prevalence of cognitive decline,^25^ in part attributed to cerebrovascular disease or uremia‐induced changes in blood–brain barrier permeability,^26^ it remains unknown whether impaired kidney function has a causal role in AD pathogenesis.^27^, ^28^ Instead, it may be more likely that impaired kidney function leads to reduced clearance of blood proteins, including those proteins measured here. Such reduced clearance would lead to an artificial inflation of these biomarker values independent of underlying AD pathology and neurodegeneration. This hypothesis is supported by our finding that even plasma levels of Aβ42, which are lower in individuals with AD, were still higher in participants with worse kidney functioning. Given that this cohort is free of stage 4 or 5 CKD, these findings are particularly noteworthy because missed diagnosis is very common in earlier stages of CKD as clinically noticeable symptoms typically do not present in these early stages. Approximately 90% of adults who meet diagnostic criteria for CKD go undiagnosed.^8^ While the rate of missed diagnosis decreases with increasing severity of CKD, the recent REVEAL‐CKD multi‐national study showed that > 70% of patients with eGFR‐confirmed stage 3 CKD were undiagnosed.^29^ In our own cohort, we found that > 88% of participants with eGFR‐defined stage 3 CKD did not self‐report kidney disease in their medical history intake form (data not shown). It is likely, therefore, that many patients seeking AD biomarker testing in the clinical setting will have undiagnosed CKD, which will confound their biomarker levels and lead to misdiagnosis of AD or related dementias. Thus, it may be beneficial for all patients undergoing plasma biomarker evaluation for AD to have contemporaneous eGFR measured to aid in interpretation.

The effect of kidney function was most pronounced for plasma NfL, which is consistent with past work linking eGFR and plasma NfL.^27^, ^30^, ^31^ We expand this prior work to show that the degree to which NfL is affected by eGFR is sufficient to cause a modification in the biomarker's ability to predict clinical outcomes. The neuropsychological outcomes most impacted by this effect modification included longitudinal trajectories of language and processing speed, both of which have been identified as key drivers of functional decline in older adults.^32^, ^33^ For neuroimaging outcomes, longitudinal atrophy of the hippocampus and expansion of the inferior lateral ventricle (representing medial temporal lobe atrophy), along with an increase in frontal lobe WMHs, were most impacted by the kidney function effect modification. While hippocampal and medial temporal lobe volumes are highly specific to AD,^34^ frontal lobe WMHs are less common in the earlier stages of AD and more likely to represent other pathological processes.^35^ These findings suggest that the effect of kidney function on the predictive accuracy of NfL is highly important as the impact is most pronounced on the ability of the biomarker to predict key, wide‐ranging clinical outcomes associated with functional independence and multiple pathologies. The reason NfL is particularly sensitive to kidney function changes is not well understood. Though speculative, it may reflect a direct effect of uremia on NfL levels through reductions in neuroprotective molecules, such as erythropoietin and vitamin D,^6^ or through comorbid conditions common to CKD that also affect NfL levels, such as diabetic peripheral neuropathy and cerebrovascular disease.^36^ While plasma NfL may provide useful prognostic and diagnostic information for many patients, our findings suggest that results should be interpreted with caution in individuals with stage 3 or greater CKD.

There are notable racial disparities in kidney disease prevalence,^37^ and we found here that there was a much higher proportion of Black participants in the stage 3 CKD group than in the other groups marked by better kidney function. Our findings of reduced biomarker performance, especially for NfL, in individuals with worse kidney function therefore have significant impacts for the equitable implementation of blood biomarkers. Black patients with memory impairment are known to experience lower rates of amyloid positivity than non‐Hispanic White patients;^38^ thus, non–amyloid‐related biomarkers, such as NfL, have been proposed as effective tools for predicting future cognitive decline in Black participants.^39^ Given the significant effects of kidney function on the predictive accuracy of plasma NfL, our findings raise suspicion of the clinical utility of this biomarker and further underscore the pressing need for more biomarker research in diverse populations.

Outside of NfL, we found interactions between eGFR and other plasma biomarkers, including GFAP, Aβ42, and p‐tau_231_, on neuropsychological and neuroimaging outcomes; however, these associations were not as prominent as our NfL results and did not withstand correction for multiple comparisons. Given the pattern observed here of weaker associations between these plasma biomarkers and many clinical outcomes in individuals with worsening kidney function, it may be expected that associations continue to attenuate in individuals with worse CKD (stage 4 or 5) not studied here. Future studies in cohorts with more significant kidney impairment would be well suited to investigate this hypothesis.

This study had several strengths. The study's longitudinal nature with a mean 6.4‐year follow up (range 1.4 to 9.7 years) offers a unique opportunity to understand how intact versus mildly compromised kidney functioning can affect prediction of future cognitive decline and brain imaging changes using blood‐based biomarkers. The comprehensive neuropsychological and neuroimaging protocols used here provide meaningful characterizations of clinical changes associated with blood‐based biomarkers. Additionally, this well‐characterized cohort allows for thorough consideration of potential confounding variables. However, the study limitations should also be discussed. This cohort is predominantly non‐Hispanic White and well educated. It is also a medically healthy cohort without participants with stage 4 or 5 CKD, thereby limiting our ability to generalize the results to participants with more severe kidney dysfunction. Kidney function was measured by a proxy, eGFR, which could be affected by other non‐renal factors (e.g., muscle mass, fluid intake). Consistent with the longitudinal nature of the study, participants with worse health status are more likely to prematurely drop out of the study; however, baseline levels of all blood‐based biomarkers and eGFR levels did not differ between those participants who continued in the study and those participants who only completed a baseline visit (data not shown). Finally, there was a risk of false positives due to the number of statistical models run; however, we corrected for this concern with a conservative FDR correction.

In sum, this study shows that kidney function, as estimated by eGFR levels, affects the levels of circulating blood proteins used as biomarkers for AD and concomitant pathologies and moderates the association between some of these blood‐based biomarkers and key clinical outcomes. As blood‐based biomarkers are more widely implemented in clinics, these findings provide key insight into how a common medical condition like CKD, which affects 38% of older adults, could contribute to misinterpretation of results.

## CONFLICT OF INTEREST STATEMENT

A.L.J. has served as an advisor for Lantheus – Diagnostic and Therapeutic Innovations. T.J.H. serves on the Scientific Advisory Board for Vivid Genomics and serves as deputy editor for *Alzheimer's and Dementia: TRCI* and senior associate editor for *Alzheimer's and Dementia*. L.T.D. serves as a consultant for Nashville Biosciences, LLC. H.Z. has served on scientific advisory boards and/or as a consultant for Abbvie, Acumen, Alector, Alzinova, ALZpath, Amylyx, Annexon, Apellis, Artery Therapeutics, AZTherapies, Cognito Therapeutics, CogRx, Denali, Eisai, LabCorp, Merry Life, Nervgen, Novo Nordisk, Optoceutics, Passage Bio, Pinteon Therapeutics, Prothena, Red Abbey Labs, reMYND, Roche, Samumed, Siemens Healthineers, Triplet Therapeutics, and Wave, and has given lectures sponsored by Alzecure, BioArctic, Biogen, Cellectricon, Fujirebio, Lilly, Novo Nordisk, Roche, and WebMD. K.B. has served as a consultant, on advisory boards, or on data monitoring committees for Abcam, Axon, Biogen, JOMDD/Shimadzu. Julius Clinical, Lilly, MagQu, Novartis, Roche Diagnostics, and Siemens Healthineers. H.Z. and K.B. are co‐founders of Brain Biomarker Solutions in Gothenburg AB, which is a part of the GU Ventures Incubator Program. C.J.B., P.Z., D.N., D.L., L.T.D., K.R.P., N.S., S.W., D.R., C.B., H.K., and K.A.G. have no conflicts of interest to disclose. Author disclosures are available in the supporting information.

## CONSENT STATEMENT

The Vanderbilt University Medical Center Institutional Review Board granted ethical approval. All participants provided informed written consent to participate in the Vanderbilt Memory and Aging Project prior to study enrollment.

## Supporting information



Supporting Information

Supporting Information

Supporting Information

Supporting Information

Supporting Information
