# Supplementary material for: Mild kidney dysfunction affects the predictive accuracy of blood‐based biomarkers for neuropsychological and neuroimaging outcomes over a 9 year follow‐up period
Source: Alzheimers Dement. 2025 Sep 19;21(9):e70651. doi: 10.1002/alz.70651 (PMC12447110; doi:10.1002/alz.70651)
Supplement: Supplementary file 1 — Supporting Information [file ALZ-21-e70651-s001.docx]

| **Supplemental Table 1. Annual Change in Neuropsychological and Imaging Outcomes Stratified by CKD Stage** | | | | | |
| --- | --- | --- | --- | --- | --- |
|  | **Combined**  **(n=304)** | **No CKD/ Stage 1**  **(n=52)** | **Stage 2**  **(n=201)** | **Stage 3**  **(n=51)** | **p-value^b^** |
| **Neuropsychological Outcomes** | | | | | |
| Boston Naming Test, total | -0.3±0.9 | -0.4±1.2 | -0.3±0.8 | -0.1±0.6 | 0.94 |
| Animal Fluency, total | -0.6±1.1 | -0.7±1.3 | -0.4±1.0 | -0.8±1.3 | **0.05** |
| Number Sequencing, seconds | 3.4±10 | 6.0±13 | 2.3±7 | 5.2±16 | 0.07 |
| Digit Symbol Coding, total | -1.6±2.8 | -1.5±2.9 | -1.7±2.9 | -1.4±2.4 | 0.77 |
| HVOT, total | -0.3±0.9 | -0.3±0.9 | -0.3±1.0 | -0.1±0.7 | 0.69 |
| Executive Function Composite, z | -0.1±0.2 | -0.1+0.2 | -0.1±0.2 | -0.1±0.2 | 0.88 |
| Episodic Memory Composite, z | -0.1±0.2 | -0.1±0.2 | -0.1±0.2 | -0.1±0.2 | 0.34 |
| Mean Follow-Up Time, years | 6.4±2.5 | 6.7±2.7 | 6.5±2.4 | 5.3±2.4 | **0.002** |
| **Brain Imaging Outcomes** | | | | | |
| AD-Signature Cortical Thickness, mm^2^ | -0.01±0.02 | -0.02±0.03 | -0.01±0.02 | -0.01±0.02 | **0.04** |
| Frontal Lobe Grey Matter, cm^3^ | -1.3±4.5 | -0.3±6.3 | -1.2±3.8 | -2.7±4.5 | **0.004** |
| Temporal Lobe Grey Matter, cm^3^ | -0.6±1.6 | -0.5±2.1 | -0.5±1.4 | -1.0±1.4 | **0.005** |
| Parietal Lobe Grey Matter, cm^3^ | -1.1±2.3 | -0.6±3.0 | -1.0±2.0 | -1.7±2.4 | **0.002** |
| Occipital Lobe Grey Matter, cm^3^ | -0.4±0.9 | -0.4±1.0 | -0.3±0.9 | -0.6±1.0 | 0.05 |
| Hippocampal Volume, cm^3^ | -0.07±0.09 | -0.08±0.11 | -0.07±0.09 | -0.09±0.08 | **0.04** |
| Inferior Lateral Ventricle Volume, cm^3^ | 0.2±0.2 | 0.2±0.2 | 0.1±0.2 | 0.2±0.2 | 0.13 |
| Frontal WMH Volume, cm^3^ | 1.1±1.5 | 1.1±2.0 | 1.1±1.4 | 1.2±1.1 | 0.06 |
| Temporal WMH Volume, cm^3^ | 0.1±0.3 | 0.2±0.5 | 0.1±0.2 | 0.2±0.5 | 0.47 |
| Parietal WMH Volume, cm^3^ | 0.6±0.8 | 0.6±1.0 | 0.5±0.8 | 0.6±0.7 | 0.17 |
| Occipital WMH Volume, cm^3^ | 0.3±0.7 | 0.3±0.6 | 0.2±0.7 | 0.4±0.8 | 0.75 |
| Mean Follow-Up Time, years | 6.0±2.5 | 6.4±2.7 | 6.2±2.5 | 5.0±2.2 | **0.01** |
| **Note.** Values denoted as mean ± standard deviation or frequency. Bold font indicates p<0.05. AD, Alzheimer’s disease; CKD, chronic kidney disease; HVOT, Hooper Visual Organization Test; MoCA, Montreal Cognitive Assessment; WMH, white matter hyperintensity. ^a^A modified FSRP score excluded points assigned to age. ^b^Kruskal-Wallis’ test was used for continuous variables, and Pearson’s chi-square test was used for categorical variables. | | | | | |
