# Supplementary material for: Mild kidney dysfunction affects the predictive accuracy of blood‐based biomarkers for neuropsychological and neuroimaging outcomes over a 9 year follow‐up period
Source: Alzheimers Dement. 2025 Sep 19;21(9):e70651. doi: 10.1002/alz.70651 (PMC12447110; doi:10.1002/alz.70651)
Supplement: Supplementary file 2 — Supporting Information [file ALZ-21-e70651-s005.docx]

| **Supplemental Table 2. Baseline Blood-Based Biomarker Associations with Cross-Sectional Clinical Outcomes** | | | | | | |
| --- | --- | --- | --- | --- | --- | --- |
|  | **β** | **95% CI** | | | **p-value** | **p-FDR** |
| **Plasma GFAP** |  |  | | |  |  |
| Boston Naming Test | -0.005 | -0.009, | -0.002 | | **0.002** | **0.01** |
| Animal Fluency | -0.006 | -0.01, | -0.0007 | | **0.03** | 0.10 |
| Number Sequencing | 0.01 | -0.009, | 0.04 | | 0.23 | 0.34 |
| Coding | -0.006 | -0.02, | 0.007 | | 0.35 | 0.45 |
| Hooper Visual Organization Test | -0.002 | -0.006, | 0.002 | | 0.16 | 0.27 |
| Executive Function Composite | -0.002 | -0.003, | -0.001 | | **<0.001** | **0.0004** |
| Episodic Memory Composite | -0.001 | -0.002, | -0.0006 | | **0.001** | **0.009** |
| AD-Signature Cortical Thickness | -0.0002 | -0.0004, | -0.0001 | | **0.0006** | **0.03** |
| Frontal Lobe Grey Matter | 0.68 | -26.77, | 28.12 | | 0.96 | 0.98 |
| Temporal Lobe Grey Matter | -4.43 | -14.85, | 5.99 | | 0.40 | 0.67 |
| Parietal Lobe Grey Matter | 5.12 | -8.93, | 19.17 | | 0.47 | 0.70 |
| Occipital Lobe Grey Matter | 1.39 | -6.37, | 9.15 | | 0.72 | 0.87 |
| Hippocampal Grey Matter | -0.31 | -1.16, | 0.54 | | 0.48 | 0.70 |
| Inferior Lateral Ventricle Volume | 2.25 | 0.96, | 3.55 | | **0.0007** | **0.03** |
| Frontal WMHs | 0.006 | -0.007, | 0.02 | | 0.34 | 0.64 |
| Temporal WMHs | 0.0001 | -0.002, | 0.002 | | 0.92 | 0.98 |
| Parietal WMHs | 0.002 | -0.005, | 0.01 | | 0.57 | 0.74 |
| Occipital WMHs | 0.002 | -0.0009, | 0.005 | | 0.16 | 0.57 |
| **Plasma NfL** |  |  | | |  |  |
| Boston Naming Test | -0.03 | -0.04, | | -0.01 | **0.001** | **0.009** |
| Animal Fluency | -0.04 | -0.07, | | -0.02 | **0.001** | **0.009** |
| Number Sequencing | 0.07 | -0.03, | | 0.18 | 0.17 | 0.29 |
| Coding | -0.04 | -0.10, | | 0.02 | 0.23 | 0.34 |
| Hooper Visual Organization Test | -0.03 | -0.04, | | -0.009 | **0.002** | **0.01** |
| Executive Function Composite | -0.006 | -0.01, | | -0.002 | **0.002** | **0.01** |
| Episodic Memory Composite | -0.004 | -0.008, | | -0.0002 | **0.04** | 0.12 |
| AD-Signature Cortical Thickness | -0.0003 | -0.0009, | | 0.0003 | 0.28 | 0.64 |
| Frontal Lobe Grey Matter | 77.80 | -49.84, | | 205.40 | 0.23 | 0.64 |
| Temporal Lobe Grey Matter | 27.53 | -20.98, | | 76.03 | 0.27 | 0.64 |
| Parietal Lobe Grey Matter | 57.26 | -7.97, | | 122.50 | 0.09 | 0.55 |
| Occipital Lobe Grey Matter | 36.02 | 0.07, | | 71.96 | **0.05** | 0.47 |
| Hippocampal Grey Matter | -3.91 | -7.86, | | 0.03 | **0.05** | 0.47 |
| Inferior Lateral Ventricle Volume | 6.52 | 0.43, | | 12.61 | **0.04** | 0.46 |
| Frontal WMHs | 0.001 | -0.06, | | 0.06 | 0.97 | 0.98 |
| Temporal WMHs | 0.003 | -0.006, | | 0.01 | 0.54 | 0.72 |
| Parietal WMHs | -0.001 | -0.04, | | 0.03 | 0.94 | 0.98 |
| Occipital WMHs | 0.007 | -0.008, | | 0.02 | 0.36 | 0.64 |
| **Plasma Aβ_42_** |  |  | | |  |  |
| Boston Naming Test | -0.004 | -0.13, | | 0.12 | 0.95 | 0.96 |
| Animal Fluency | 0.007 | -0.20, | | 0.22 | 0.95 | 0.96 |
| Number Sequencing | 0.07 | -0.76, | | 0.90 | 0.87 | 0.93 |
| Coding | 0.19 | -0.31, | | 0.69 | 0.45 | 0.53 |
| Hooper Visual Organization Test | -0.08 | -0.21, | | 0.05 | 0.20 | 0.31 |
| Executive Function Composite | -0.03 | -0.06, | | 0.005 | 0.10 | 0.20 |
| Episodic Memory Composite | -0.01 | -0.04, | | 0.02 | 0.43 | 0.51 |
| AD-Signature Cortical Thickness | 0.005 | 0.00002, | | 0.01 | **0.05** | 0.47 |
| Frontal Lobe Grey Matter | 1320 | 311.3, | | 2329 | **0.01** | 0.31 |
| Temporal Lobe Grey Matter | 186.4 | -200.3, | | 573.1 | 0.34 | 0.64 |
| Parietal Lobe Grey Matter | 613.4 | 95.62, | | 1131 | **0.02** | 0.40 |
| Occipital Lobe Grey Matter | 162.6 | -125, | | 450.2 | 0.27 | 0.64 |
| Hippocampal Grey Matter | -5.82 | -37.44, | | 25.8 | 0.72 | 0.87 |
| Inferior Lateral Ventricle Volume | 0.31 | -48.58, | | 49.2 | 0.99 | 0.99 |
| Frontal WMHs | -0.23 | -0.71, | | 0.25 | 0.35 | 0.64 |
| Temporal WMHs | -0.03 | -0.10, | | 0.05 | 0.50 | 0.72 |
| Parietal WMHs | -0.20 | -0.49, | | 0.08 | 0.16 | 0.57 |
| Occipital WMHs | -0.05 | -0.16, | | 0.07 | 0.43 | 0.70 |
| **Plasma p-tau_231_** |  |  | | |  |  |
| Boston Naming Test | -0.05 | -0.12, | | 0.02 | 0.17 | 0.29 |
| Animal Fluency | 0.01 | -0.11, | | 0.13 | 0.87 | 0.93 |
| Number Sequencing | 0.03 | -0.45, | | 0.52 | 0.90 | 0.95 |
| Coding | 0.16 | -0.13, | | 0.45 | 0.27 | 0.38 |
| Hooper Visual Organization Test | -0.07 | -0.15, | | 0.003 | 0.06 | 0.14 |
| Executive Function Composite | -0.02 | -0.04, | | -0.001 | **0.04** | 0.12 |
| Episodic Memory Composite | -0.02 | -0.04, | | -0.0008 | **0.04** | 0.12 |
| AD-Signature Cortical Thickness | -0.0006 | -0.004, | | 0.002 | 0.66 | 0.81 |
| Frontal Lobe Grey Matter | 92.14 | -504.2, | | 688.4 | 0.76 | 0.89 |
| Temporal Lobe Grey Matter | -111.80 | -338, | | 114.4 | 0.33 | 0.64 |
| Parietal Lobe Grey Matter | 24.16 | -281.4, | | 329.7 | 0.88 | 0.94 |
| Occipital Lobe Grey Matter | 85.67 | -82.66, | | 254 | 0.32 | 0.64 |
| Hippocampal Grey Matter | -6.74 | -25.23, | | 11.74 | 0.47 | 0.70 |
| Inferior Lateral Ventricle Volume | 13.66 | -14.90, | | 42.22 | 0.35 | 0.64 |
| Frontal WMHs | -0.13 | -0.41, | | 0.15 | 0.37 | 0.64 |
| Temporal WMHs | -0.02 | -0.06, | | 0.02 | 0.36 | 0.64 |
| Parietal WMHs | -0.12 | -0.28, | | 0.05 | 0.17 | 0.57 |
| Occipital WMHs | -0.009 | -0.08, | | 0.06 | 0.79 | 0.90 |
| **Note.** Models were adjusted for age, sex, education, race/ethnicity, *APOE*-ε4 status, Framingham Stroke Risk Profile, and cognitive status. β Indicates the degree of change in outcomes per 1 unit increase in the respective blood-based biomarker. Bold font indicates p<0.05. Aβ_42_, amyloid beta 42; AD, Alzheimer’s disease; *APOE*-ε4, apolipoprotein E ε4; FDR, false discovery rate; GFAP, glial fibrillary acidic protein; NfL, neurofilament light; p-tau_,_ phosphorylated tau; WMHs, white matter hyperintensities. | | | | | | |
