# Supplementary material for: Mild kidney dysfunction affects the predictive accuracy of blood‐based biomarkers for neuropsychological and neuroimaging outcomes over a 9 year follow‐up period
Source: Alzheimers Dement. 2025 Sep 19;21(9):e70651. doi: 10.1002/alz.70651 (PMC12447110; doi:10.1002/alz.70651)
Supplement: Supplementary file 3 — Supporting Information [file ALZ-21-e70651-s003.docx]

| **Supplemental Table 3. Blood-Based Biomarker x eGFR Interactions on Cross-Sectional Clinical Outcomes** | | | | | | | | | | | | | | | | | | | | | | | | | | | |  |
| --- | --- | --- | --- | --- | --- | --- | --- | --- | --- | --- | --- | --- | --- | --- | --- | --- | --- | --- | --- | --- | --- | --- | --- | --- | --- | --- | --- | --- |
|  | **Interaction Model (n=333)** | | | | | | | **No CKD/Stage 1 (n=57)** | | | | | | | **Stage 2 CKD (n=217)** | | | | | | | **Stage 3 CKD (n=59)** | | | | | |  |
|  | **β** | | **95% CI** | | | **p** | | **β** | | **95% CI** | | | **p** | | **β** | | **95% CI** | | | **p** | | **β** | | **95% CI** | | | **p** |  |
| **Plasma GFAP** | | | | | | | |  | | | | | | |  | | | | | | |  | |  | | |  |  |
| BNT | -3.1E-5 | -2.1E-4, | | 1.5E-4 | 0.74 | | -1.5E-3 | | -9.3E-3, | | 6.2E-3 | 0.69 | | -8.2E-3 | | -1.2E-2, | | -4.0E-3 | ***0.0002*** | | -2.7E-3 | | -1.2E-2, | | 6.3E-3 | 0.55 | | |
| Animal Fluency | 1.7E-4 | -1.3E-4, | | 4.7E-4 | 0.28 | | 3.4E-3 | | -9.8E-3, | | 1.7E-2 | 0.60 | | -1.1E-2 | | -1.9E-2, | | -3.9E-3 | ***0.003*** | | -5.7E-3 | | -1.9E-2, | | 7.9E-3 | 0.40 | | |
| Number Sequencing | 1.2E-4 | -1.1E-3, | | 1.3E-3 | 0.84 | | -1.5E-2 | | -5.3E-2, | | 2.4E-2 | 0.44 | | 3.2E-2 | | 5.5E-4, | | 6.4E-2 | **0.05** | | -7.4E-3 | | -6.0E-2, | | 4.6E-2 | 0.78 | | |
| Coding | 4.3E-4 | -2.8E-4, | | 1.1E-3 | 0.24 | | 2.3E-2 | | -9.3E-3, | | 5.5E-2 | 0.16 | | -1.1E-2 | | -2.9E-2, | | 7.0E-3 | 0.23 | | -1.4E-2 | | -4.3E-2, | | 1.6E-2 | 0.36 | | |
| HVOT | -9.5E-5 | -2.8E-4, | | 9.1E-5 | 0.32 | | -8.9E-4 | | -9.9E-3, | | 8.1E-3 | 0.84 | | -4.0E-3 | | -8.4E-3, | | 3.2E-4 | 0.07 | | -9.7E-4 | | -9.7E-3, | | 7.8E-3 | 0.83 | | |
| EF Composite | 2.0E-5 | -2.4E-5, | | 6.3E-5 | 0.37 | | 9.1E-4 | | -9.8E-4, | | 2.8E-3 | 0.34 | | -2.9E-3 | | -3.8E-3, | | -1.9E-3 | ***<0.0001*** | | -1.6E-3 | | -3.7E-3, | | 5.7E-4 | 0.15 | | |
| Memory Composite | -1.1E-5 | -5.6E-5, | | 3.4E-5 | 0.64 | | -6.3E-4 | | -2.6E-3, | | 1.4E-3 | 0.53 | | -2.2E-3 | | -3.4E-3, | | -1.1E-3 | ***<0.0001*** | | 1.0E-4 | | -1.6E-3, | | 1.8E-3 | 0.90 | | |
| AD-Signature | -7.0E-6 | -1.4E-5, | | -9.3E-8 | **0.05** | | -3.7E-4 | | -6.3E-4, | | -9.6E-5 | **0.01** | | -2.2E-4 | | -4.0E-4, | | -3.9E-5 | **0.02** | | -1.5E-4 | | -4.2E-4, | | 1.3E-4 | 0.29 | | |
| Frontal Lobe GM | 6.2E-2 | -1.9E+0, | | 2.0E+0 | 0.95 | | 2.2E+1 | | -4.0E+1, | | 8.3E+1 | 0.48 | | 1.7E+1 | | -3.3E+1, | | 6.8E+1 | 0.50 | | 2.7E+1 | | -5.9E+1, | | 1.1E+2 | 0.53 | | |
| Temporal Lobe GM | -6.2E-1 | -1.5E+0, | | 2.3E-1 | 0.15 | | -1.5E+1 | | -5.1E+1, | | 2.1E+1 | 0.41 | | 4.7E+0 | | -1.8E+1, | | 2.7E+1 | 0.68 | | 2.0E+1 | | -1.2E+1, | | 5.2E+1 | 0.22 | | |
| Parietal Lobe GM | -2.9E-1 | -1.3E+0, | | 7.1E-1 | 0.57 | | -1.2E+0 | | -3.6E+1, | | 3.4E+1 | 0.95 | | 2.1E+1 | | -5.2E+0, | | 4.8E+1 | 0.11 | | 1.3E+1 | | -2.5E+1, | | 5.0E+1 | 0.49 | | |
| Occipital Lobe GM | -3.0E-1 | -9.3E-1, | | 3.2E-1 | 0.34 | | 4.3E+0 | | -2.0E+1, | | 2.8E+1 | 0.72 | | 8.0E+0 | | -8.3E+0, | | 2.4E+1 | 0.33 | | 1.1E+1 | | -1.4E+1, | | 3.7E+1 | 0.38 | | |
| Hippocampus | -2.7E-2 | -7.7E-2, | | 2.3E-2 | 0.30 | | -1.8E+0 | | -4.1E+0, | | 4.4E-1 | 0.11 | | 8.2E-1 | | -4.0E-1, | | 2.0E+0 | 0.19 | | 1.2E-1 | | -2.1E+0, | | 2.4E+0 | 0.91 | | |
| ILV | 1.8E-2 | -5.2E-2, | | 8.8E-2 | 0.61 | | 3.6E+0 | | 8.6E-1, | | 6.3E+0 | **0.01** | | 2.2E+0 | | 4.4E-1, | | 3.9E+0 | **0.01** | | 2.4E+0 | | -8.9E-1, | | 5.7E+0 | 0.15 | | |
| Frontal WMHs | 3.0E-4 | -3.9E-4, | | 1.0E-3 | 0.39 | | 2.8E-2 | | -1.5E-2, | | 7.0E-2 | 0.19 | | 3.0E-3 | | -1.3E-2, | | 1.9E-2 | 0.72 | | 1.2E-2 | | -1.4E-2, | | 3.7E-2 | 0.36 | | |
| Temporal WMHs | 7.8E-5 | -2.8E-5, | | 1.8E-4 | 0.15 | | 3.1E-3 | | -5.0E-3, | | 1.1E-2 | 0.44 | | -1.4E-5 | | -2.3E-3, | | 2.3E-3 | 0.99 | | 4.0E-4 | | -1.8E-3, | | 2.6E-3 | 0.71 | | |
| Parietal WMHs | 2.6E-4 | -1.4E-4, | | 6.7E-4 | 0.20 | | 1.6E-2 | | -7.6E-3, | | 4.0E-2 | 0.18 | | -1.1E-5 | | -1.0E-2, | | 1.0E-2 | 1.00 | | 5.4E-3 | | -6.8E-3, | | 1.8E-2 | 0.38 | | |
| Occipital WMHs | 1.7E-4 | 1.7E-6, | | 3.4E-4 | **0.05** | | 9.4E-3 | | 8.2E-4, | | 1.8E-2 | **0.03** | | 2.4E-3 | | -1.7E-3, | | 6.5E-3 | 0.25 | | 4.4E-4 | | -5.8E-3, | | 6.7E-3 | 0.89 | | |
| **Plasma NfL** | | | | | | | |  | | | | | | |  | | | | | | |  | |  | | |  |  |
| BNT | -3.8E-4 | -1.2E-3, | | 4.5E-4 | 0.37 | | -6.2E-2 | | -1.2E-1, | | -5.8E-4 | 0.05 | | -4.0E-2 | | -6.7E-2, | | -1.2E-2 | ***0.005*** | | -2.0E-2 | | -4.9E-2, | | 8.6E-3 | 0.17 | | |
| Animal Fluency | 9.8E-5 | -1.3E-3, | | 1.5E-3 | 0.89 | | -6.2E-2 | | -1.7E-1, | | 4.6E-2 | 0.26 | | -6.9E-2 | | -1.2E-1, | | -2.0E-2 | **0.006** | | -5.0E-2 | | -9.3E-2, | | -7.6E-3 | **0.02** | | |
| Number Sequencing | 2.6E-3 | -3.0E-3, | | 8.1E-3 | 0.36 | | -1.3E-2 | | -3.3E-1, | | 3.0E-1 | 0.94 | | 1.1E-1 | | -1.0E-1, | | 3.1E-1 | 0.31 | | 7.0E-2 | | -1.0E-1, | | 2.4E-1 | 0.42 | | |
| Coding | -3.8E-3 | -7.1E-3, | | -5.5E-4 | **0.02** | | -9.7E-2 | | -3.6E-1, | | 1.7E-1 | 0.47 | | -6.1E-2 | | -1.8E-1, | | 5.6E-2 | 0.30 | | 3.6E-3 | | -9.4E-2, | | 1.0E-1 | 0.94 | | |
| HVOT | -9.1E-4 | -1.7E-3, | | -6.4E-5 | **0.04** | | -1.1E-1 | | -1.8E-1, | | -4.5E-2 | **0.002** | | -2.3E-2 | | -5.1E-2, | | 5.0E-3 | 0.11 | | -1.8E-2 | | -4.7E-2, | | 9.7E-3 | 0.19 | | |
| EF Composite | -1.7E-4 | -3.7E-4, | | 3.4E-5 | 0.10 | | -2.9E-3 | | -1.9E-2, | | 1.3E-2 | 0.71 | | -1.6E-2 | | -2.2E-2, | | -9.2E-3 | ***<0.0001*** | | -3.4E-3 | | -1.1E-2, | | 3.6E-3 | 0.33 | | |
| Memory Composite | -2.0E-4 | -4.1E-4, | | 6.6E-6 | 0.06 | | -1.1E-2 | | -2.7E-2, | | 5.5E-3 | 0.19 | | -1.2E-2 | | -2.0E-2, | | -4.7E-3 | ***0.002*** | | 1.5E-4 | | -5.4E-3, | | 5.7E-3 | 0.96 | | |
| AD-Signature | -5.3E-5 | -8.4E-5, | | -2.2E-5 | ***0.001*** | | -2.6E-3 | | -4.8E-3, | | -3.7E-4 | **0.02** | | -7.1E-4 | | -2.0E-3, | | 5.6E-4 | 0.27 | | -2.5E-5 | | -9.2E-4, | | 8.7E-4 | 0.96 | | |
| Frontal Lobe GM | 1.5E+0 | -7.3E+0, | | 1.0E+1 | 0.74 | | -8.3E+1 | | -5.9E+2, | | 4.3E+2 | 0.74 | | -5.1E+1 | | -3.8E+2, | | 2.7E+2 | 0.76 | | -2.8E+1 | | -3.1E+2, | | 2.6E+2 | 0.84 | | |
| Temporal Lobe GM | -2.3E+0 | -6.2E+0, | | 1.5E+0 | 0.24 | | -3.1E+2 | | -5.9E+2, | | -2.3E+1 | **0.04** | | -5.1E+1 | | -1.9E+2, | | 9.2E+1 | 0.48 | | 3.9E+1 | | -6.7E+1, | | 1.5E+2 | 0.46 | | |
| Parietal Lobe GM | 1.4E+0 | -3.2E+0, | | 5.9E+0 | 0.55 | | -7.6E+1 | | -3.6E+2, | | 2.1E+2 | 0.59 | | 5.6E+1 | | -1.2E+2, | | 2.3E+2 | 0.52 | | -6.7E+0 | | -1.3E+2, | | 1.2E+2 | 0.91 | | |
| Occipital Lobe GM | -2.6E-1 | -3.1E+0, | | 2.6E+0 | 0.86 | | -5.9E+1 | | -2.5E+2, | | 1.4E+2 | 0.55 | | 2.2E+1 | | -8.3E+1, | | 1.3E+2 | 0.68 | | 1.6E+0 | | -8.4E+1, | | 8.7E+1 | 0.97 | | |
| Hippocampus | -1.5E-1 | -3.8E-1, | | 7.5E-2 | 0.19 | | -3.0E+1 | | -4.7E+1, | | -1.3E+1 | **0.001** | | -6.1E+0 | | -1.4E+1, | | 1.7E+0 | 0.13 | | 3.0E-1 | | -7.2E+0, | | 7.8E+0 | 0.94 | | |
| ILV | 5.9E-1 | 2.7E-1, | | 9.1E-1 | ***0.0003*** | | 3.5E+1 | | 1.4E+1, | | 5.7E+1 | **0.002** | | 1.8E+1 | | 7.0E+0, | | 2.9E+1 | **0.002** | | -4.7E+0 | | -1.6E+1, | | 6.4E+0 | 0.40 | | |
| Frontal WMHs | 6.4E-4 | -2.5E-3, | | 3.8E-3 | 0.69 | | 1.2E-1 | | -2.3E-1, | | 4.8E-1 | 0.48 | | 2.1E-2 | | -8.5E-2, | | 1.3E-1 | 0.70 | | -8.2E-3 | | -9.2E-2, | | 7.6E-2 | 0.85 | | |
| Temporal WMHs | 5.5E-4 | 6.6E-5, | | 1.0E-3 | **0.03** | | 6.8E-2 | | 5.1E-3, | | 1.3E-1 | **0.03** | | 4.3E-3 | | -1.0E-2, | | 1.9E-2 | 0.57 | | 1.1E-3 | | -6.2E-3, | | 8.4E-3 | 0.76 | | |
| Parietal WMHs | 9.8E-4 | -8.9E-4, | | 2.9E-3 | 0.30 | | 9.9E-2 | | -9.9E-2, | | 3.0E-1 | 0.32 | | 2.7E-2 | | -3.7E-2, | | 9.2E-2 | 0.40 | | -3.9E-3 | | -4.4E-2, | | 3.7E-2 | 0.85 | | |
| Occipital WMHs | 4.2E-4 | -3.6E-4, | | 1.2E-3 | 0.29 | | 4.2E-2 | | -3.1E-2, | | 1.2E-1 | 0.25 | | 1.6E-2 | | -1.0E-2, | | 4.2E-2 | 0.23 | | 6.1E-3 | | -1.4E-2, | | 2.7E-2 | 0.55 | | |
| **Plasma Aβ_42_** | | | | | | | |  | | | | | | |  | | | | | | |  | |  | | |  |  |
| BNT | -8.3E-3 | -1.7E-2, | | 4.2E-4 | 0.06 | | -5.3E-1 | | -9.7E-1, | | -8.3E-2 | **0.02** | | 2.7E-2 | | -1.2E-1, | | 1.7E-1 | 0.71 | | 2.1E-1 | | -1.3E-1, | | 5.4E-1 | 0.22 | | |
| Animal Fluency | -1.4E-3 | -1.6E-2, | | 1.3E-2 | 0.85 | | -1.2E-1 | | -9.2E-1, | | 6.8E-1 | 0.77 | | 4.0E-3 | | -2.5E-1, | | 2.6E-1 | 0.98 | | 1.1E-1 | | -4.1E-1, | | 6.2E-1 | 0.69 | | |
| Number Sequencing | 3.0E-2 | -2.8E-2, | | 8.7E-2 | 0.31 | | -1.5E+0 | | -3.8E+0, | | 7.4E-1 | 0.18 | | 7.9E-1 | | -2.8E-1, | | 1.9E+0 | 0.15 | | -1.4E+0 | | -3.4E+0, | | 5.6E-1 | 0.16 | | |
| Coding | 1.3E-2 | -2.1E-2, | | 4.7E-2 | 0.45 | | 2.0E+0 | | 7.5E-2, | | 3.9E+0 | **0.04** | | -1.5E-1 | | -7.5E-1, | | 4.6E-1 | 0.64 | | 1.1E+0 | | 5.7E-2, | | 2.2E+0 | **0.04** | | |
| HVOT | 5.5E-3 | -3.4E-3, | | 1.4E-2 | 0.22 | | 2.5E-1 | | -2.8E-1, | | 7.9E-1 | 0.35 | | -1.0E-1 | | -2.5E-1, | | 4.5E-2 | 0.17 | | 5.6E-3 | | -3.3E-1, | | 3.4E-1 | 0.97 | | |
| EF Composite | 1.2E-3 | -8.7E-4, | | 3.4E-3 | 0.25 | | 5.4E-2 | | -6.0E-2, | | 1.7E-1 | 0.35 | | -3.2E-2 | | -6.7E-2, | | 3.4E-3 | 0.08 | | -8.9E-3 | | -9.2E-2, | | 7.4E-2 | 0.83 | | |
| Memory Composite | 2.4E-3 | 2.1E-4, | | 4.5E-3 | **0.03** | | 7.3E-2 | | -4.6E-2, | | 1.9E-1 | 0.22 | | -2.4E-4 | | -4.1E-2, | | 4.0E-2 | 0.99 | | -3.7E-2 | | -1.0E-1, | | 2.6E-2 | 0.24 | | |
| AD-Signature | -1.5E-4 | -4.8E-4, | | 1.9E-4 | 0.38 | | -6.2E-4 | | -1.9E-2, | | 1.8E-2 | 0.95 | | 4.1E-3 | | -1.7E-3, | | 9.9E-3 | 0.17 | | 9.2E-3 | | -2.3E-3, | | 2.1E-2 | 0.11 | | |
| Frontal Lobe GM | -9.4E+0 | -1.0E+2, | | 8.3E+1 | 0.84 | | 4.5E+2 | | -3.4E+3, | | 4.3E+3 | 0.81 | | 1.3E+3 | | -3.5E+2, | | 3.0E+3 | 0.12 | | -4.4E+2 | | -3.7E+3, | | 2.8E+3 | 0.79 | | |
| Temporal Lobe GM | -5.4E+0 | -4.6E+1, | | 3.5E+1 | 0.79 | | 2.6E+2 | | -2.0E+3, | | 2.5E+3 | 0.82 | | 6.4E+1 | | -6.9E+2, | | 8.2E+2 | 0.87 | | -4.1E+2 | | -1.6E+3, | | 8.2E+2 | 0.51 | | |
| Parietal Lobe GM | -2.3E+1 | -7.1E+1, | | 2.5E+1 | 0.34 | | -6.1E+2 | | -2.7E+3, | | 1.5E+3 | 0.57 | | 7.5E+2 | | -1.5E+2, | | 1.6E+3 | 0.10 | | -3.8E+2 | | -1.8E+3, | | 1.1E+3 | 0.60 | | |
| Occipital Lobe GM | -3.0E+0 | -3.3E+1, | | 2.7E+1 | 0.85 | | -4.5E+2 | | -1.9E+3, | | 1.0E+3 | 0.54 | | 1.0E+2 | | -4.5E+2, | | 6.5E+2 | 0.71 | | -3.5E+2 | | -1.3E+3, | | 6.2E+2 | 0.47 | | |
| Hippocampus | 1.2E+0 | -1.2E+0, | | 3.6E+0 | 0.34 | | 2.9E+1 | | -1.2E+2, | | 1.7E+2 | 0.69 | | 7.6E+0 | | -3.4E+1, | | 4.9E+1 | 0.72 | | -6.5E+1 | | -1.5E+2, | | 1.9E+1 | 0.13 | | |
| ILV | -1.7E+0 | -5.2E+0, | | 1.7E+0 | 0.32 | | -9.1E+1 | | -2.7E+2, | | 8.8E+1 | 0.31 | | -1.9E+1 | | -7.8E+1, | | 4.1E+1 | 0.53 | | 6.1E+1 | | -6.7E+1, | | 1.9E+2 | 0.34 | | |
| Frontal WMHs | -2.8E-2 | -6.1E-2, | | 5.8E-3 | 0.11 | | -3.6E+0 | | -6.0E+0, | | -1.2E+0 | **0.004** | | 1.1E-1 | | -4.5E-1, | | 6.6E-1 | 0.70 | | -9.3E-2 | | -1.1E+0, | | 8.7E-1 | 0.85 | | |
| Temporal WMHs | -1.5E-3 | -6.6E-3, | | 3.6E-3 | 0.56 | | -5.0E-1 | | -9.8E-1, | | -2.5E-2 | **0.04** | | 6.3E-2 | | -1.4E-2, | | 1.4E-1 | 0.11 | | -6.5E-2 | | -1.5E-1, | | 1.7E-2 | 0.12 | | |
| Parietal WMHs | -4.6E-3 | -2.4E-2, | | 1.5E-2 | 0.64 | | -1.9E+0 | | -3.2E+0, | | -5.2E-1 | **0.008** | | 1.0E-1 | | -2.3E-1, | | 4.4E-1 | 0.54 | | -3.5E-1 | | -8.1E-1, | | 1.1E-1 | 0.13 | | |
| Occipital WMHs | 1.3E-3 | -6.8E-3, | | 9.4E-3 | 0.75 | | -4.6E-1 | | -9.9E-1, | | 6.2E-2 | 0.08 | | 5.1E-2 | | -8.7E-2, | | 1.9E-1 | 0.47 | | -1.3E-1 | | -3.6E-1, | | 1.1E-1 | 0.28 | | |
| **Plasma p-tau_231_** | | | | | | | |  | | | | | | |  | | | | | | |  | |  | | |  |  |
| BNT | -3.8E-3 | -7.7E-3, | | 1.4E-4 | 0.06 | | -1.8E-1 | | -3.7E-1, | | 3.3E-3 | 0.05 | | -7.9E-2 | | -1.7E-1, | | 1.6E-2 | 0.10 | | 9.8E-2 | | -6.9E-2, | | 2.7E-1 | 0.24 | | |
| Animal Fluency | 1.1E-3 | -5.5E-3, | | 7.7E-3 | 0.74 | | 1.4E-1 | | -1.9E-1, | | 4.6E-1 | 0.41 | | -2.5E-2 | | -1.9E-1, | | 1.4E-1 | 0.77 | | 3.4E-2 | | -2.2E-1, | | 2.9E-1 | 0.79 | | |
| Number Sequencing | 1.7E-2 | -9.0E-3, | | 4.3E-2 | 0.20 | | -4.1E-1 | | -1.4E+0, | | 5.3E-1 | 0.38 | | 6.5E-1 | | -4.9E-2, | | 1.3E+0 | 0.07 | | -8.4E-1 | | -1.8E+0, | | 1.3E-1 | 0.09 | | |
| Coding | 2.1E-3 | -1.4E-2, | | 1.8E-2 | 0.79 | | 6.9E-1 | | -9.0E-2, | | 1.5E+0 | 0.08 | | -7.1E-2 | | -4.7E-1, | | 3.3E-1 | 0.73 | | 5.0E-1 | | -4.2E-2, | | 1.0E+0 | 0.07 | | |
| HVOT | -1.3E-3 | -5.3E-3, | | 2.7E-3 | 0.53 | | -1.7E-1 | | -3.8E-1, | | 5.0E-2 | 0.13 | | -6.1E-2 | | -1.6E-1, | | 3.5E-2 | 0.21 | | -7.3E-3 | | -1.7E-1, | | 1.6E-1 | 0.93 | | |
| EF Composite | -1.1E-4 | -1.1E-3, | | 8.5E-4 | 0.83 | | 1.5E-2 | | -3.2E-2, | | 6.1E-2 | 0.53 | | -2.7E-2 | | -5.0E-2, | | -4.1E-3 | **0.02** | | -1.6E-2 | | -5.7E-2, | | 2.5E-2 | 0.43 | | |
| Memory Composite | 8.6E-4 | -1.2E-4, | | 1.8E-3 | 0.08 | | 3.4E-2 | | -1.4E-2, | | 8.3E-2 | 0.16 | | -2.1E-2 | | -4.7E-2, | | 5.4E-3 | 0.12 | | -3.5E-2 | | -6.5E-2, | | -4.9E-3 | **0.02** | | |
| AD-Signature | -1.1E-4 | -2.6E-4, | | 3.7E-5 | 0.14 | | -3.6E-3 | | -1.1E-2, | | 4.2E-3 | 0.35 | | -2.6E-3 | | -6.6E-3, | | 1.4E-3 | 0.19 | | 3.2E-3 | | -2.0E-3, | | 8.4E-3 | 0.22 | | |
| Frontal Lobe GM | -3.3E+1 | -7.5E+1, | | 8.2E+0 | 0.12 | | 2.1E+2 | | -1.3E+3, | | 1.7E+3 | 0.79 | | -4.2E+1 | | -1.2E+3, | | 1.1E+3 | 0.94 | | 1.0E+3 | | -5.7E+2, | | 2.6E+3 | 0.20 | | |
| Temporal Lobe GM | -9.9E+0 | -2.8E+1, | | 8.5E+0 | 0.29 | | 6.8E-1 | | -9.1E+2, | | 9.1E+2 | 1.00 | | -8.7E+1 | | -5.8E+2, | | 4.1E+2 | 0.73 | | 1.9E+2 | | -4.2E+2, | | 8.0E+2 | 0.54 | | |
| Parietal Lobe GM | -1.7E+1 | -3.9E+1, | | 4.2E+0 | 0.11 | | -2.4E+2 | | -1.1E+3, | | 6.2E+2 | 0.58 | | 1.3E+2 | | -4.6E+2, | | 7.2E+2 | 0.67 | | 3.1E+2 | | -4.0E+2, | | 1.0E+3 | 0.38 | | |
| Occipital Lobe GM | -1.1E+1 | -2.4E+1, | | 2.7E+0 | 0.12 | | 2.2E+1 | | -5.7E+2, | | 6.2E+2 | 0.94 | | 6.0E+1 | | -3.0E+2, | | 4.2E+2 | 0.74 | | 4.0E+2 | | -7.6E+1, | | 8.7E+2 | 0.10 | | |
| Hippocampus | 1.3E-1 | -9.7E-1, | | 1.2E+0 | 0.82 | | -1.1E+1 | | -6.9E+1, | | 4.7E+1 | 0.71 | | 1.1E+0 | | -2.6E+1, | | 2.8E+1 | 0.94 | | 2.5E+0 | | -4.0E+1, | | 4.5E+1 | 0.91 | | |
| ILV | -1.6E-1 | -1.7E+0, | | 1.4E+0 | 0.84 | | 2.7E+1 | | -4.6E+1, | | 9.9E+1 | 0.46 | | 1.7E+1 | | -2.2E+1, | | 5.6E+1 | 0.40 | | 1.9E+1 | | -4.5E+1, | | 8.2E+1 | 0.56 | | |
| Frontal WMHs | -2.3E-3 | -1.7E-2, | | 1.3E-2 | 0.77 | | -7.6E-1 | | -1.8E+0, | | 2.9E-1 | 0.15 | | 1.0E-1 | | -2.6E-1, | | 4.6E-1 | 0.58 | | -5.6E-2 | | -5.4E-1, | | 4.2E-1 | 0.81 | | |
| Temporal WMHs | 3.4E-4 | -2.0E-3, | | 2.7E-3 | 0.77 | | -1.4E-1 | | -3.4E-1, | | 5.8E-2 | 0.16 | | 4.2E-2 | | -8.5E-3, | | 9.2E-2 | 0.10 | | -3.7E-2 | | -7.8E-2, | | 2.8E-3 | 0.07 | | |
| Parietal WMHs | 1.2E-3 | -7.7E-3, | | 1.0E-2 | 0.79 | | -4.8E-1 | | -1.1E+0, | | 1.1E-1 | 0.11 | | 1.2E-1 | | -1.0E-1, | | 3.4E-1 | 0.30 | | -2.3E-1 | | -4.5E-1, | | -1.1E-2 | **0.04** | | |
| Occipital WMHs | 1.1E-3 | -2.6E-3, | | 4.8E-3 | 0.56 | | -7.7E-2 | | -3.0E-1, | | 1.5E-1 | 0.49 | | 7.0E-2 | | -2.1E-2, | | 1.6E-1 | 0.13 | | -5.1E-2 | | -1.7E-1, | | 6.6E-2 | 0.39 | | |
| **Note.** Models were adjusted for age, sex, education, race/ethnicity, *APOE*-ε4 status, Framingham Stroke Risk Profile, and cognitive status. β Indicates the degree of change in outcomes per 1 unit increase in the respective blood-based biomarker. Bold font indicates p<0.05. Italic font indicates significant associations persisted after FDR-correction. Aβ_42_, amyloid beta 42; AD, Alzheimer’s disease; *APOE*-ε4, apolipoprotein E ε4; BNT, Boston Naming Test; CKD, chronic kidney disease; EF, executive functioning; eGFR, estimated glomerular filtration rate; FDR, false discovery rate; GFAP, glial fibrillary acidic protein; GM, grey matter; HVOT, Hooper Visual Organization Test; ILV, inferior lateral ventricle; NfL, neurofilament light; p-tau_,_ phosphorylated tau; WMHs, white matter hyperintensities. | | | | | | | | | | | | | | | | | | | | | | | | | | | |  |
