# Supplementary material for: Mild kidney dysfunction affects the predictive accuracy of blood‐based biomarkers for neuropsychological and neuroimaging outcomes over a 9 year follow‐up period
Source: Alzheimers Dement. 2025 Sep 19;21(9):e70651. doi: 10.1002/alz.70651 (PMC12447110; doi:10.1002/alz.70651)
Supplement: Supplementary file 4 — Supporting Information [file ALZ-21-e70651-s002.docx]

| **Supplemental Table 4. Baseline Blood-Based Biomarker Associations with Longitudinal Clinical Outcomes** | | | | | |
| --- | --- | --- | --- | --- | --- |
|  | **β** | **95% CI** | | **p-value** | **p-FDR** |
| **Plasma GFAP** |  |  | |  |  |
| Boston Naming Test | -0.003 | -0.004 | -0.002 | **<0.0001** | **<0.0001** |
| Animal Fluency | -0.002 | -0.003 | -0.0009 | **0.0003** | **0.0007** |
| Number Sequencing | 0.01 | 0.005 | 0.02 | **0.001** | **0.003** |
| Coding | -0.007 | -0.009 | -0.005 | **<0.0001** | **<0.0001** |
| Hooper Visual Organization Test | -0.003 | -0.003 | -0.002 | **<0.0001** | **<0.0001** |
| Executive Function Composite | -0.0004 | -0.0006 | -0.0003 | **<0.0001** | **<0.0001** |
| Episodic Memory Composite | -0.0004 | -0.0005 | -0.0003 | **<0.0001** | **<0.0001** |
| AD-Signature Cortical Thickness | 0.00001 | -0.00003 | 0.000006 | 0.19 | 0.27 |
| Frontal Lobe Grey Matter | -4.57 | -8.65 | -0.50 | **0.03** | 0.06 |
| Temporal Lobe Grey Matter | -3.22 | -4.54 | -1.90 | **<0.0001** | **<0.0001** |
| Parietal Lobe Grey Matter | -3.37 | -5.32 | -1.41 | **0.0008** | **0.004** |
| Occipital Lobe Grey Matter | -1.70 | -2.51 | -0.88 | **0.0001** | **0.0004** |
| Hippocampal Grey Matter | -0.30 | -0.40 | -0.22 | **<0.0001** | **<0.0001** |
| Inferior Lateral Ventricle Volume | 0.78 | 0.57 | 0.99 | **<0.0001** | **<0.0001** |
| Frontal WMHs | 0.003 | 0.002 | 0.004 | **<0.0001** | **0.0001** |
| Temporal WMHs | 0.0002 | -0.000004 | 0.0004 | 0.05 | 0.10 |
| Parietal WMHs | 0.002 | 0.0008 | 0.002 | **0.0001** | **0.0005** |
| Occipital WMHs | 0.0006 | 0.0002 | 0.001 | **0.003** | **0.01** |
| **Plasma NfL** |  |  | |  |  |
| Boston Naming Test | -0.01 | -0.02, | -0.009 | **<0.0001** | **<0.0001** |
| Animal Fluency | -0.02 | -0.02, | -0.01 | **<0.0001** | **<0.0001** |
| Number Sequencing | 0.13 | 0.07, | 0.18 | **<0.0001** | **<0.0001** |
| Coding | -0.05 | -0.06, | -0.03 | **<0.0001** | **<0.0001** |
| Hooper Visual Organization Test | -0.02 | -0.02, | -0.01 | **<0.0001** | **<0.0001** |
| Executive Function Composite | -0.004 | -0.005, | -0.003 | **<0.0001** | **<0.0001** |
| Episodic Memory Composite | -0.003 | -0.004, | -0.002 | **<0.0001** | **<0.0001** |
| AD-Signature Cortical Thickness | -0.0001 | -0.0003, | 0.00001 | 0.07 | 0.13 |
| Frontal Lobe Grey Matter | -26.99 | -53.96, | -0.02 | **0.05** | 0.10 |
| Temporal Lobe Grey Matter | -20.25 | -29.04, | -11.46 | **<0.0001** | **0.0001** |
| Parietal Lobe Grey Matter | -20.11 | -33.17, | -7.05 | **0.003** | **0.009** |
| Occipital Lobe Grey Matter | -10.95 | -16.39, | -5.51 | **0.0001** | **0.0005** |
| Hippocampal Grey Matter | -1.70 | -2.28, | -1.11 | **<0.0001** | **<0.0001** |
| Inferior Lateral Ventricle Volume | 4.05 | 2.93, | 5.17 | **<0.0001** | **<0.0001** |
| Frontal WMHs | 0.02 | 0.008, | 0.02 | **<0.0001** | **0.0003** |
| Temporal WMHs | 0.001 | -0.0004, | 0.003 | 0.16 | 0.24 |
| Parietal WMHs | 0.007 | 0.003, | 0.01 | **0.002** | **0.006** |
| Occipital WMHs | 0.004 | 0.0009, | 0.007 | **0.009** | **0.02** |
| **Plasma Aβ_42_** |  |  | |  |  |
| Boston Naming Test | 0.008 | -0.03, | 0.04 | 0.66 | 0.74 |
| Animal Fluency | 0.01 | -0.02, | 0.05 | 0.46 | 0.59 |
| Number Sequencing | 0.10 | -0.22, | 0.41 | 0.55 | 0.66 |
| Coding | -0.01 | -0.09, | 0.06 | 0.78 | 0.80 |
| Hooper Visual Organization Test | 0.02 | -0.003, | 0.05 | 0.09 | 0.14 |
| Executive Function Composite | 0.001 | -0.005, | 0.007 | 0.74 | 0.77 |
| Episodic Memory Composite | 0.002 | -0.004, | 0.007 | 0.54 | 0.66 |
| AD-Signature Cortical Thickness | 0.00004 | -0.0006, | 0.0007 | 0.89 | 0.90 |
| Frontal Lobe Grey Matter | -139.40 | -290.30, | 11.37 | 0.07 | 0.12 |
| Temporal Lobe Grey Matter | -22.27 | -69.32, | 24.77 | 0.35 | 0.45 |
| Parietal Lobe Grey Matter | -80.98 | -153.30, | -8.64 | **0.03** | 0.06 |
| Occipital Lobe Grey Matter | -29.20 | -59.30, | 0.91 | 0.06 | 0.11 |
| Hippocampal Grey Matter | -0.19 | -3.88, | 3.50 | 0.92 | 0.92 |
| Inferior Lateral Ventricle Volume | -1.77 | -10.48, | 6.93 | 0.69 | 0.72 |
| Frontal WMHs | 0.006 | -0.05, | 0.06 | 0.83 | 0.85 |
| Temporal WMHs | 0.002 | -0.006, | 0.01 | 0.64 | 0.68 |
| Parietal WMHs | 0.01 | -0.02, | 0.04 | 0.53 | 0.58 |
| Occipital WMHs | 0.005 | -0.009, | 0.02 | 0.48 | 0.55 |
| **Plasma p-tau_231_** |  |  | |  |  |
| Boston Naming Test | -0.05 | -0.07, | -0.03 | **<0.0001** | **<0.0001** |
| Animal Fluency | -0.04 | -0.06, | -0.02 | **0.0003** | **0.0007** |
| Number Sequencing | 0.33 | 0.14, | 0.52 | **0.0007** | **0.002** |
| Coding | -0.08 | -0.12, | -0.03 | **0.001** | **0.002** |
| Hooper Visual Organization Test | -0.03 | -0.04, | -0.01 | **0.002** | **0.004** |
| Executive Function Composite | -0.005 | -0.009, | -0.002 | **0.003** | **0.006** |
| Episodic Memory Composite | -0.006 | -0.009, | -0.003 | **0.0002** | **0.0005** |
| AD-Signature Cortical Thickness | -0.0003 | -0.0007, | 0.0001 | 0.16 | 0.24 |
| Frontal Lobe Grey Matter | -21.45 | -113.40, | 70.50 | 0.65 | 0.69 |
| Temporal Lobe Grey Matter | -20.09 | -49.04, | 8.87 | 0.17 | 0.26 |
| Parietal Lobe Grey Matter | -20.76 | -65.21, | 23.69 | 0.36 | 0.45 |
| Occipital Lobe Grey Matter | -10.74 | -29.42, | 7.95 | 0.26 | 0.36 |
| Hippocampal Grey Matter | -4.23 | -6.44, | -2.03 | **0.0002** | **0.0009** |
| Inferior Lateral Ventricle Volume | 11.86 | 6.86, | 16.85 | **<0.0001** | **<0.0001** |
| Frontal WMHs | 0.04 | 0.01, | 0.07 | **0.006** | **0.02** |
| Temporal WMHs | 0.002 | 0.001, | 0.004 | **0.001** | **0.005** |
| Parietal WMHs | 0.004 | 0.002, | 0.006 | **0.0005** | **0.003** |
| Occipital WMHs | 0.002 | 0.0005, | 0.004 | **0.01** | **0.03** |
| **Note.** Models were adjusted for age, sex, education, race/ethnicity, *APOE*-ε4 status, Framingham Stroke Risk Profile, and cognitive status. β Indicates the degree of change in outcomes per 1 unit increase in the respective blood-based biomarker. Bold font indicates p<0.05. Aβ_42_, amyloid beta 42; AD, Alzheimer’s disease; FDR, false discovery rate; GFAP, glial fibrillary acidic protein; NfL, neurofilament light; p-tau_,_ phosphorylated tau; WMHs, white matter hyperintensities. | | | | | |
